# Multimodal measures of spontaneous brain activity reveal both common and divergent patterns of cortical functional organization

**DOI:** 10.21203/rs.3.rs-2823802/v1

**Published:** 2023-04-26

**Authors:** Hadi Vafaii, Francesca Mandino, Gabriel Desrosiers-Grégoire, David O’Connor, Xilin Shen, Xinxin Ge, Peter Herman, Fahmeed Hyder, Xenophon Papademetris, Mallar Chakravarty, Michael C. Crair, R. Todd Constable, Evelyn MR. Lake, Luiz Pessoa

**Affiliations:** 1 Department of Physics, University of Maryland, College Park, MD, 20742, USA; 2 Department of Psychology, University of Maryland, College Park, MD, 20742, USA; 3 Department of Electrical and Computer Engineering, University of Maryland, College Park, MD, 20742, USA; 4 Department of Radiology and Biomedical Imaging, Yale School of Medicine, New Haven, CT, 06520, USA; 5 Department of Biomedical Engineering, Yale University, New Haven, CT, 06511, USA; 6 Department of Neuroscience, Yale School of Medicine, New Haven, CT, 06510, USA; 7 Kavli Institute for Neuroscience, Yale School of Medicine, New Haven, CT, 06510, USA; 8 Department of Ophthalmology and Visual Science, Yale School of Medicine, New Haven, CT, 06510, USA; 9 Department of Biomedical Engineering, Yale University, New Haven, CT, 06520, USA; 10 Department of Neurosurgery, Yale School of Medicine, New Haven, CT, 06510, USA; 11 Comp. Brain Anatomy Laboratory, Cerebral Imaging Center, Douglas Mental Health Univ. Institute, Montreal, QC, H4H 1R3, Canada; 12 Integrated Program in Neuroscience, McGill University, Montreal, QC, H3A 0G4, Canada; 13 Department of Psychiatry, McGill University, Montreal, QC, H3A 0G4, Canada; 14 Department of Biological and Biomedical Engineering, McGill University, Montreal, QC, H3A 0G4, Canada; 15 Department of Physiology, School of Medicine, University of California San Francisco, San Francisco, CA, 94143, USA

## Abstract

Large-scale functional networks have been characterized in both rodent and human brains, typically by analyzing fMRI-BOLD signals. However, the relationship between fMRI-BOLD and underlying neural activity is complex and incompletely understood, which poses challenges to interpreting network organization obtained using this technique. Additionally, most work has assumed a disjoint functional network organization (i.e., brain regions belong to one and only one network). Here, we employed wide-field Ca^2+^ imaging simultaneously with fMRI-BOLD in mice expressing GCaMP6f in excitatory neurons. We determined cortical networks discovered by each modality using a mixed-membership algorithm to test the hypothesis that functional networks are overlapping rather than disjoint. Our results show that multiple BOLD networks are detected via Ca^2+^ signals; there is considerable network overlap (both modalities); networks determined by low-frequency Ca^2+^ signals are only modestly more similar to BOLD networks; and, despite similarities, important differences are detected across modalities (e.g., brain region “network diversity”). In conclusion, Ca^2+^ imaging uncovered overlapping functional cortical organization in the mouse that reflected several, but not all, properties observed with fMRI-BOLD signals.

## Introduction

Brains show evidence of functional organization across spatiotemporal scales, from synapses to the whole organ, which varies between individuals, over time, as well as with injury or disease. Understanding the principles that govern brain organization enables their use as clinical indices. Closing knowledge gaps requires work in humans and model species, across scales, and using complementary sources of image contrast. Here, we focus on large-scale systems (i.e., networks), as a deeper understanding of their characteristics stands to have broad prognostic and diagnostic utility, in part because they can be assessed with noninvasive imaging methods that are applicable in human subjects.

Much of what we know and can access about large-scale systems, especially in humans, comes from the blood-oxygenation-level-dependent (BOLD) contrast obtained with functional magnetic resonance imaging (fMRI). Recent and growing evidence shows that measures of large-scale systems obtained with fMRI-BOLD (or proximal optical measures of hemoglobin) are, to an extent, reflective of neural activity [1–5]. Yet, despite important progress, the relationship between fMRI-BOLD and underlying neural activity is complex and incompletely understood [6–8], which poses several challenges to interpreting network organization obtained using this technique [9–13].

A powerful tool for investigating the functional organization of large-scale networks is wide-field fluorescence imaging in mouse models bearing genetically encoded calcium (Ca^2+^ ) sensitive indicators [14, 15]. Critically, Ca^2+^ imaging affords a large field of view covering much of the mouse cortical mantle, and provides image contrast that is a more direct measure of neural activity than BOLD. Applied with fMRI-BOLD (or BOLD-like measures), Ca^2+^ imaging can reveal the neural component captured by the BOLD signal [1, 2, 5, 16–18]. Here, we leverage a novel simultaneous multimodal framework, BOLD-fMRI and Ca^2+^ imaging [1], to determine both cross-modal convergent and divergent features of large-scale functional networks.

As in previous studies using similar [3, 16] or the same experimental approach [1], we examine functional connectivity, a widely employed measure of inter-regional synchrony, to define and characterize large-scale brain networks. Importantly, we consider networks as having *overlapping*, rather than disjoint, functional organization. Many complex systems, including biological, technological, and social ones, are inherently overlapping (nodes participate in multiple communities or clusters) rather than *disjoint* (each node belongs to a single community) [19–21]. In the brain, overlap means that regions participate across multiple networks (to varying degrees), consistent with the notion that functionally flexible regions can contribute to multiple brain processes [22–25]. Although evidence for overlap in human brain networks has accrued based on multiple analysis techniques applied to BOLD-fMRI data [25–28], it is unclear whether the putative overlapping organization is driven, at least in part, by the nature of BOLD signals. To the best of our knowledge, the potentially overlapping functional organization of cortical networks has not been tested in animal models, where fMRI-BOLD can be obtained together with Ca^2+^ signals that exhibit greater spatiotemporal resolution and capture neural activity more directly.

**Figure 1:**
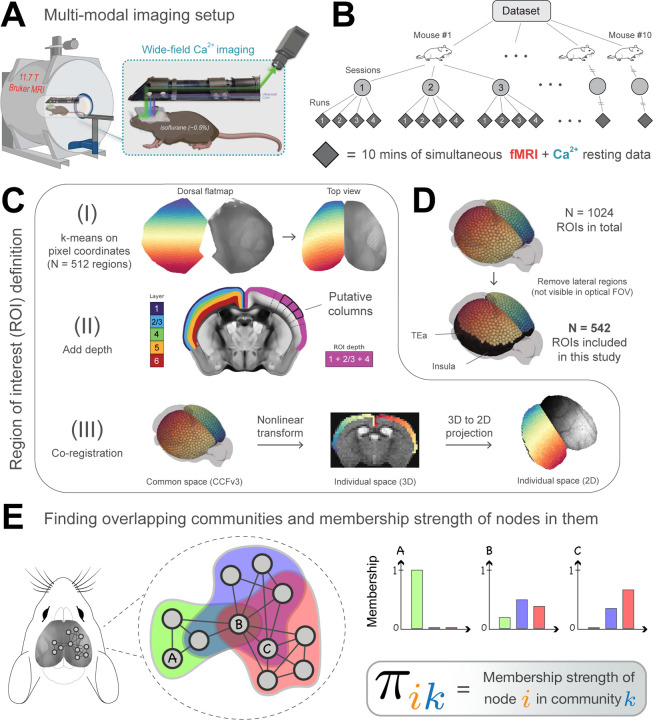
Experimental setup and overlapping community analysis. **(A)** Simultaneous fMRI-BOLD and wide-field Ca^2+^ imaging [1]. Ca^2+^ data are background-corrected (illustrated by three colored wavelengths; Methods) **(B)** Hierarchical data structure. *N* = 10 mice, scanned across 3 longitudinal sessions, with 4 runs per session, each lasting 10 minutes. **(C)** Definition of ROIs within the Allen Mouse Brain Common Coordinate Framework (CCFv3) [35]. (I) Division of the mouse dorsal flatmap into *N* = 1024 spatially homogeneous ROIs. (II) Add depth by following streamlines normal to the cortical surface. The resulting ROIs are “column-like”. (III) Transform ROIs from common space into 3D and 2D individual spaces (Methods). Dorsal flatmap, layer masks, and columnar streamlines from CCFv3. **(D)** Analyses were restricted to ROIs that appeared in the Ca^2+^ imaging FOV after multimodal co-registration (Methods). Lateral areas including the insula and temporal association areas were excluded. **(E)** We applied a mixed-membership stochastic blockmodel algorithm to estimate overlapping communities [29]. *Membership strength* (values between 0 and 1) quantifies the affiliation strength of a node in a network. Here, node *A* belongs only to the green community, node *B* belongs to all three communities with varying strengths, and node *C* belongs to the blue and red communities with varying strengths.

Here, we use highly-sampled simultaneously recorded wide-field Ca^2+^ and fMRI-BOLD data to resolve whether functional networks discovered with BOLD are also detected with Ca^2+^ imaging, while determining their overlapping organization (Figure 1). We use a Bayesian generative algorithm that estimates the membership strength of a given brain region to all networks [25, 27, 29]. Importantly, this approach also allows detection of disjoint organization in a data-driven manner. In addition, region-level properties are quantified including node degree [30, 31] and diversity [32–34], while a wide range of parameters are explored to test the robustness of our findings (Table 1).

Overall, we find that overlapping network organization is robustly detected in simultaneously recorded wide-field Ca^2+^ and fMRI-BOLD data regardless of the parameters selected. Evidence of rich overlapping organization advances our fundamental understanding of cortical brain organization, helping to further validate the neural origins of clinically accessible fMRI-BOLD network organization.

## Results

Mice (N=10) expressing GCaMP6f in excitatory neurons underwent simultaneous wide-field Ca^2+^ and BOLD-fMRI, as described previously by us ([1]; Methods, Figure 1A). Animals were lightly anesthetized (0.50%−0.75% isoflurane) and head-fixed. Data were collected at each of 3 longitudinal sessions; each session contained 4 runs, each lasting 10 minutes (Figure 1B) for a total of 1,200 minutes of data.

BOLD data (acquisition rate 1 *Hz*, Methods) were processed using RABIES (Rodent Automated BOLD Improvement of EPI Sequences) [36–38] and high-pass filtered [39] (0.01–0.5 *Hz*). Given that Ca^2+^ and BOLD signals are maximally correlated when Ca^2+^ is temporally band-passed to match BOLD [1, 2, 17], and the “lowpass” nature of the BOLD signal [40–42], we investigated network measures within a slow (BOLD-matched) and fast (0.5–5 *Hz*) Ca^2+^ frequency range (herein, ${\text{Ca}}_{slow}^{2+}$ and ${\text{Ca}}_{fast}^{2+}$). Ca^2+^ data were acquired at an effective background-corrected rate of 10 *Hz* and processed as described by us previously [43]. Critically, we collected both GCaMP-sensitive and GCaMP-insensitive optical measurements for the removal of background fluorescence and hemoglobin signals from the Ca^2+^ data ([1, 43–45]; Methods).

To build functional networks, a common set of regions of interest (ROIs) were defined (Figure 1C; Methods). To relate 3D BOLD and 2D Ca^2+^ data, we adopted the CCFv3 space for the mouse brain provided by the Allen Institute for Brain Sciences [35]. ROIs covered most of the cortex. Areas not well captured in the wide-field Ca^2+^ imaging FOV were excluded (Figure 1D). Correlation matrices were computed for each acquisition run using pairwise Pearson correlation. Matrices were binarized by retaining the top *d*% strongest edges (Table 1). We used a mixed-membership stochastic blockmodel algorithm [46] that can generate overlapping (or disjoint) networks [25, 27, 29]. The algorithm determines *membership* values for each ROI, with one value per network (Figure 1E). Membership values sum to 1 across networks, which allows these values to be interpreted as probabilities. Overlapping networks, and by extension membership values, were computed at the level of *runs*, then averaged across sessions to determine an animal-level result. Random-effects group analysis was evaluated based on animal-level estimates and variability. Results in the main text are from 542 ROIs and *d* = 15%.

### Traditional cortical organization captured by overlapping network solutions

**Figure 2:**
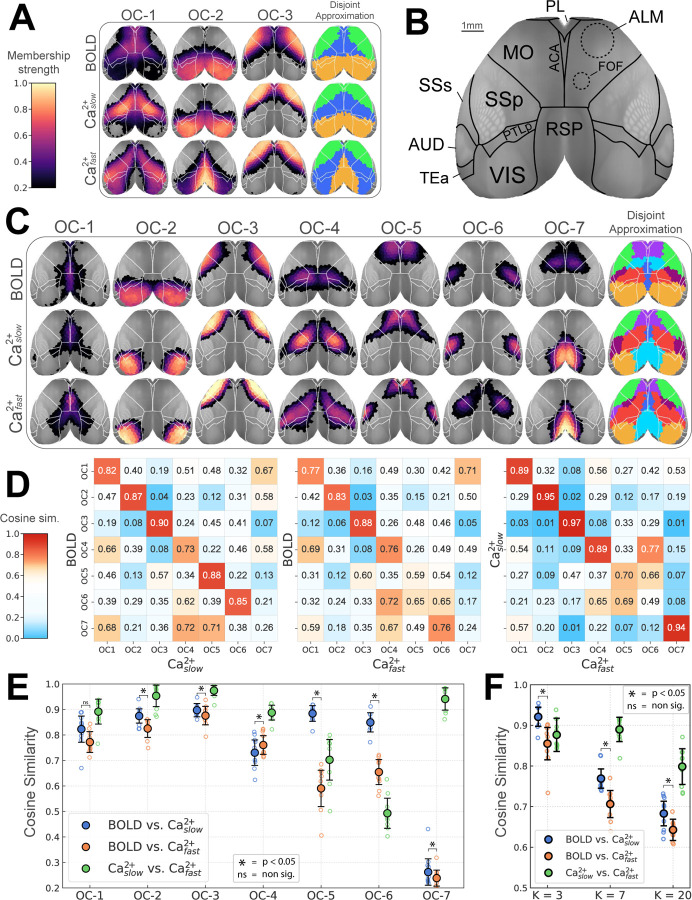
Overlapping functional networks of the mouse cortex. **(A)** Decomposition based on 3 networks. Color scale indicates membership strengths (Figure 1E). The disjoint approximation is obtained by taking each region’s maximum membership value. **(B)** Cortical areas (top view) as defined in the CCFv3 Allen reference atlas [35]. In addition, dashed lines approximately correspond to functionally defined subregions in the secondary motor area [55, 59]. **(C)** Decomposition with 7 networks. **(D)** Network similarity based on cosine similarity (1 = identical, 0.5 = “orthogonal”, 0 = perfectly dissimilar or “inversely correlated”). Color scale emphasizes similarities and strong dissimilarities. **(E)** Diagonal elements of matrices in D are plotted. Empty circles correspond to individual animals; the large solid circle is the group average. **(F)** Overall similarity collapsing across networks as a function of number of networks (3, 7, and 20). **(E-F)** Comparison of BOLD and Ca^2+^ networks (*p* < 0.05, Holm-Bonferroni corrected). Error bars are 95% confidence intervals based on hierarchical bootstrap. Abbreviations: OC, overlapping community; ACA, anterior cingulate area; ALM, anterior lateral motor cortex; FOF, frontal orienting field; MO, somatomotor areas; PL, prelimbic area; PTLp, posterior parietal association areas; RSP, retrosplenial area; SSp, primary somatosensory area; SSs, supplemental somatosensory area; VIS, visual areas. See also Figure S1, Figure S2, Figure S3, and Figure S4.

Here, *network* is used interchangeably with *overlapping community*, as is *node* with *region*. Existing work has shown decomposition of the mouse cortex into as few as 2–3 networks [4, 33, 47], but 7–10 is more typical [37, 48–50]. We explored a range numbers of networks (3, 7, and 20). Our 3-network solution captured previously observed systems, namely, the visual (overlapping community 2, OC-2) and somatomotor (OC-3), as well as a large system (OC-1) that included territories previously classified as the mouse “default network” [49, 51–53] (Figure 2A). To facilitate comparisons to standard *disjoint* algorithms, we forced a disjoint version of our solutions by assigning each region to the network with the largest community membership value.

With 7 networks, well-defined visual and somatomotor networks (OC-2 and OC-3, respectively) were again identified [37, 50], alongside additional systems covering bilateral and well-defined cortical territories (Figure 2C). OC-1 encompassed medial areas including the cingulate cortex but also extended more laterally. OC-4 spanned from medial to lateral areas, including somatosensory cortex. For both ${\text{Ca}}_{fast}^{2+}$ and ${\text{Ca}}_{slow}^{2+}$, OC-4 also included the frontal orienting field (FOF), a possible homolog of the frontal eye field in primates [54–58]. OC-5 largely overlapped with the anterior lateral motor area, a region involved in motor planning [44, 59–61]; notably, for ${\text{Ca}}_{fast}^{2+}$ this network also included the supplementary somatosensory area. OC-6 overlapped with the barrel field for BOLD and ${\text{Ca}}_{slow}^{2+}$, but captured the upper limb somatosensory cortex for ${\text{Ca}}_{fast}^{2+}$. Finally, OC-7 was very different for BOLD and Ca^2+^ signals; for BOLD, it was centered around FOF, and for both Ca^2+^ signals it was centered around the retrosplenial cortex.

The 20-network solution is shown in Figure S1, which revealed finer spatial networks that were again bilateral (like the 3- and 7-network solutions). Notably, even with 20 networks, the FOF did not appear as a separate network for either Ca^2+^ signal, in contrast to BOLD. In sum, across solutions (3, 7, and 20 networks), recognized functional organization (established brain regions, functional networks, and a high degree of bilateral symmetry) was uncovered using our algorithm across imaging modalities.

### Intermodal network organization similarity

For 7 networks, BOLD, ${\text{Ca}}_{slow}^{2+}$, and ${\text{Ca}}_{fast}^{2+}$ were quantitatively compared (Figure 2D) to test the hypothesis that band-pass filtering Ca^2+^ to match BOLD leads to greater intermodal agreement. The comparison was based on cosine similarity (1 indicates identical organization, 0.5 indicates “orthogonal/unrelated” organization, and 0 indicates perfectly “opposite” organization). The similarity between BOLD and ${\text{Ca}}_{slow}^{2+}$ networks was relatively high (> 0.73), except for OC-7 (0.26), a network that was evident in both Ca^2+^ conditions but not captured by BOLD. In comparison, BOLD and ${\text{Ca}}_{fast}^{2+}$ similarity was generally lower but still relatively high for OC-1 to OC-4 (> 0.77), though modest for OC-5 and OC-6 (0.59 and 0.65, respectively). Overall, band-pass filtering Ca^2+^ seemed to have a modest network-dependent impact when comparing network territories across modalities.

To generate a summary metric, we collapsed across networks to generate an overall index of similarity. As expected [1–3], BOLD and ${\text{Ca}}_{slow}^{2+}$ solutions were more similar than BOLD and ${\text{Ca}}_{fast}^{2+}$ (*p* < 0.05, permutation test, Holm–Bonferroni corrected). This result was stable across 3, 7, and 20 networks, data processing parameters (Figure S3), including *d*%, and number of ROIs (Figure S2).

### Cortical networks show prominent overlapping organization

All results are described at the group-level. However, we confirmed that the basic organization of the 7-network solution was observed at the individual level (Figure S4). Thus, group-level properties, including network overlap, are *not* driven by the process of performing group analysis.

**Figure 3:**
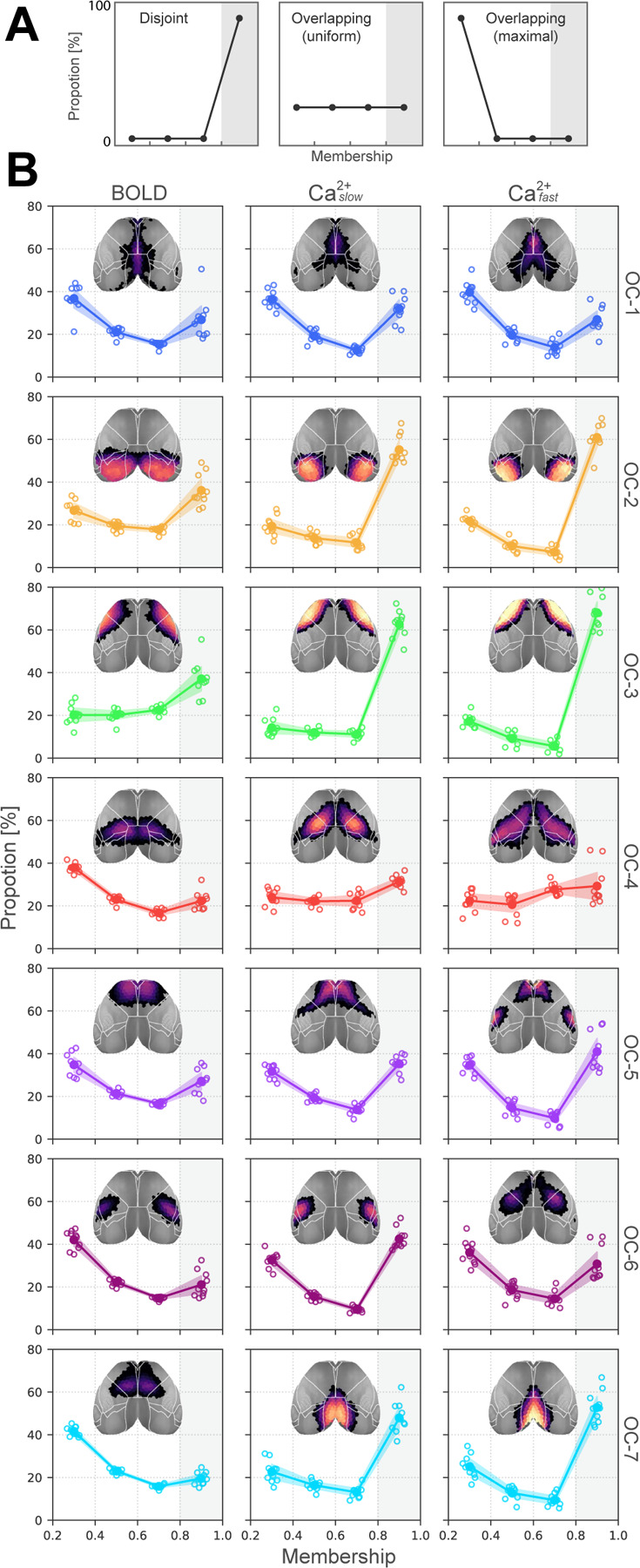
Distribution of membership values. **(A)** Three illustrative distributions. Left, disjoint organization; Middle, overlapping with uniform membership values; Right, completely overlapping with no mid-range or strong memberships. **(B)** Membership distributions computed from multimodal data indicate substantial overlapping organization. Note that the y-axis is capped at 80%, indicating that none of the networks are truly disjoint. Empty circles correspond to individual animals; large solid circles are the group average. Abbreviations: OC, overlapping community. Compare with Figure S5 for membership distributions obtained from synthetic graphs with known ground truth overlap.

To quantify overlapping organization, we examined the distribution of membership values across networks. Membership values range 0–1, and sum to 1 across networks. Thus, a disjoint organization would be characterized by all regions having high membership values for a single network (a “right-peaked” distribution; Figure 3A, left). Importantly, this outcome is observed with our algorithm when synthetic, disjoint data are simulated (see Figure S5). In contrast, a roughly uniform distribution of membership values would correspond to a network whose regions affiliate with multiple networks with varying strengths (Figure 3A, middle). Finally, extreme overlap would be when regions tend to not affiliate with any network very strongly (Figure 3A, right).

To examine membership distributions, we considered the range (0.2, 1.0]. Membership values < 0.2 were not considered so as to *conservatively* characterize network overlap. The last bin contained values > 0.8 based on our results from simulated synthetic graphs with known ground truth [62]. Specifically, we found that disjoint synthetic networks had membership values concentrated in the range (0.8, 1.0] (Figure S5). In contrast, actual data, across conditions (BOLD, ${\text{Ca}}_{slow}^{2+}$, and ${\text{Ca}}_{fast}^{2+}$), showed that no more than 60% of brain regions within any network were within this range (> 0.8). The least overlapping networks were the visual and somatomotor (OC-2 and OC-3) for all conditions, and the retrosplenial network (OC-7) for the two Ca^2+^ conditions. Networks with the greatest amount of overlap were OC-1 and OC-4 (Figure 3B), which included the cingulate cortex (OC-1) as well as medial and lateral areas, including somatosensory cortex (OC-4) and the FOF (OC-4, Ca^2+^ ).

**Figure 4:**
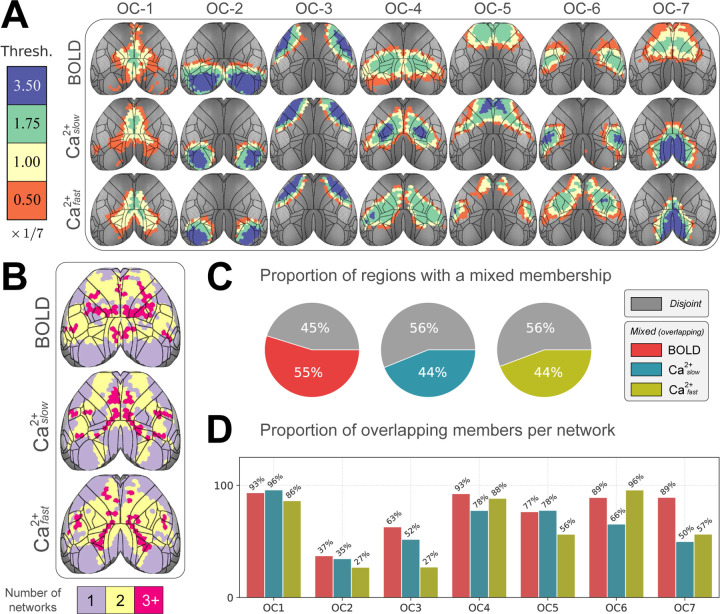
Quantifying overlap extent. **(A)** Membership values binned by statistical thresholding. Bins were incremented by 1/7 (for the 7 network solution). Blue (membership > 3.5 × 1/7) indicates regions with disjoint-like network affiliation. At the opposite end of the spectrum, orange (> 0.05 × 1/7) indicates regions affiliated with multiple networks. Contour lines correspond to regional divisions in the Allen reference atlas (Figure S1B). **(B)** Collapsing across networks. Using a statistical threshold of 1/7 to determine if a region affiliated with a network, we counted the number of networks each region “belonged to”. Most regions belonged to more than one network. **(C)** We defined a global *overlap score* as the ratio of overlapping regions divided by the total number of regions. **(D)** Overlap score at the network level shows that regions that affiliate with more than one network are spread across networks and present in both BOLD as well as ${\text{Ca}}_{fast}^{2+}$ and ${\text{Ca}}_{slow}^{2+}$. Abbreviations: OC, overlapping community.

To visualize network overlap, we binned membership strength into four categories. Because we considered 7 networks, bin thresholds were multiples of 1/7 (all statistics are FDR corrected). Based on this representation, we observed that overlapping network organization was arranged in a spatially coherent fashion that showed a tiered pattern of nested membership (Figure 4A). To quantify overlap across networks, if a region had a membership value statistically greater than 1/7 for a given network, we classified it as “belonging to” to that network. We then summed the number of networks to which regions belonged (Figure 4B). By this definition, approximately 50% of brain regions belonged to more than one network (Figure 4C). Further, brain regions belonging to more than one network were distributed across networks (Figure 4D). In even the most disjoint-like cases (OC-2 and OC-3), > 25% of regions affiliated significantly with more than one network across all conditions.

### Membership diversity reveals intermodal differences

**Figure 5:**
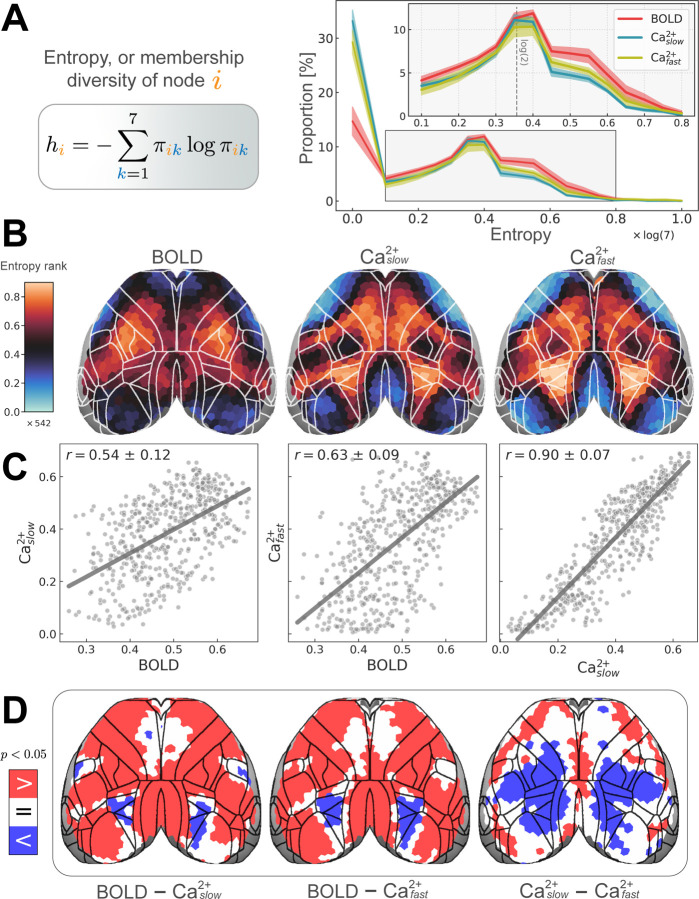
Regional entropy, or membership diversity. **(A)** Left: equation for Shannon entropy (Methods). Values are normalized [0, 1]. *h*_*i*_ = 0 if a node *i* belongs to a single network (is disjoint); *h*_*i*_ = 1 if a node belongs to all networks with equal strength (is maximally overlapping). Right: distribution of entropies for all regions. The peak at *h* = 0, corresponds to disjoint regions. The second peak at *h* ≈ log(2) corresponds to regions with membership values of 0.5 for two networks and 0 elsewhere. **(B)** Spatial distribution of regional entropies rank-ordered (total of 542 regions) to facilitate comparisons across conditions (BOLD, ${\text{Ca}}_{slow}^{2+}$, and ${\text{Ca}}_{fast}^{2+}$). The non-rank-ordered version is shown in Figure S6A. Unimodal areas such as visual and somatomotor areas have low entropy (cool colors), whereas transmodal regions have high entropy (hot colors). **(C)** Entropy was positively (Pearson) correlated across modalities (variability obtained based on hierarchical bootstrapping; Methods). **(D)** Differences in entropy between conditions quantified by subtracting each pair of conditions. Permutation testing revealed BOLD > Ca^2+^ in most regions, except for some frontal areas where BOLD = Ca^2+^, and higher visual areas where BOLD < Ca^2+^ (left and middle). ${\text{Ca}}_{slow}^{2+}$ exhibited a large territory of regions with entropy < ${\text{Ca}}_{fast}^{2+}$ (right).

The preceding analyses showed clear evidence for extensive overlapping organization in the mouse cortex across imaging modalities and frequency bands. The characteristics of this overlapping organization were further quantified using (normalized) Shannon entropy, a continuous measure of *membership diversity* computed from regional membership values (Methods, Figure 5A, left). A region that belongs to all networks with equal membership strengths will have maximal diversity. Conversely, a region that belongs to a single network will have minimal diversity. Thus, membership diversity is indicative of a region’s multi-functionality and/or involvement in multiple processes.

The distribution of membership diversity values across all regions is shown in Figure 5A (right). The peak near zero captures a group of regions, approximately 30% for ${\text{Ca}}_{slow}^{2+}$ and ${\text{Ca}}_{fast}^{2+}$, and 15% for BOLD, that are primarily associated with one network. Beyond this peak, the majority of regions displayed values more or less along a continuum, with a second smaller peak (at approximately 0.35) with regions affiliated with two networks (dashed line in Figure 5A, right inset). For visualization purposes, we rank-ordered membership diversity values to inspect the overall pattern across conditions (BOLD, ${\text{Ca}}_{slow}^{2+}$, and ${\text{Ca}}_{fast}^{2+}$; Figure 5B; for the non-rank-ordered version, see Figure S6A). The resulting patterns revealed modest agreement between BOLD and both Ca^2+^ conditions, and especially strong agreement between the two Ca^2+^ frequency bands. To quantify this agreement, we (Pearson) correlated membership diversity values: between BOLD and ${\text{Ca}}_{slow}^{2+}$ : *r* = 0.54 ± 0.11; BOLD and ${\text{Ca}}_{fast}^{2+}$ : *r* = 0.63 ± 0.09; and ${\text{Ca}}_{slow}^{2+}$ and ${\text{Ca}}_{fast}^{2+}$ : *r* = 0.90 ± 0.07 (Figure 5C). Contrary to expectations, measures of BOLD membership diversity were *not* more similar to those obtained from ${\text{Ca}}_{slow}^{2+}$ relative to between BOLD and ${\text{Ca}}_{fast}^{2+}$ (Figure 5C).

We also identified regions that showed significant differences in membership diversity magnitude between conditions by subtracting each pair of measures (Figure 5D; FDR corrected). Spatially broad differences (BOLD versus both Ca^2+^ frequency bands) were observed. Diversity was consistently larger for BOLD compared to both Ca^2+^ conditions, except for two bilateral sectors that showed the opposite pattern (one in higher-order visual areas and one, for ${\text{Ca}}_{slow}^{2+}$ only, in a primary somatosensory area). This final observation was made alongside ${\text{Ca}}_{fast}^{2+}$ exhibiting a large territory of regions with higher membership diversity than ${\text{Ca}}_{slow}^{2+}$. Overall, differences were observed despite similar proportions across modalities for values above 0.1 (Figure 5A, right).

For a comparison between membership diversity (entropy) and *participation coefficient*, a measure commonly used to quantify link diversity [32–34, 63, 64], see Figure S6. We found membership diversities and participation coefficients to be in good agreement for BOLD: *r* = 0.71 ± 0.09, and ${\text{Ca}}_{slow}^{2+}$ : *r* = 0.77 ± 0.12, and to be more weakly related for ${\text{Ca}}_{fast}^{2+}$ : *r* = 0.47 ± 0.28.

### Region degree is substantially different across modalities

**Figure 6:**
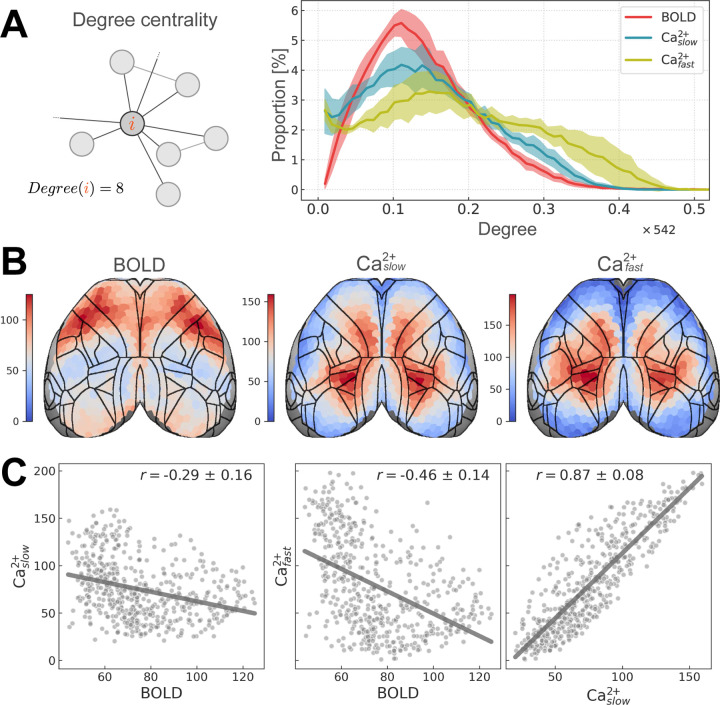
Regional degree. **(A)** Left: schematic of regional degree. Right: distribution of regional degree normalized by number of brain regions (total of 542). **(B)** Regional degree visualized on the cortex. Densely connected regions have high degree (hot colors), whereas sparsely connected regions have low degree (cool colors). BOLD and Ca^2+^ conditions show opposing spatial patterns. **(C)** Similarity between conditions is quantified using (Pearson) correlation. BOLD and Ca^2+^ are negatively correlated whereas Ca^2+^ conditions are highly positively correlated (variability obtained based on hierarchical bootstrapping; Methods).

*Degree* is a measure of centrality that quantifies the number of functional connections of a region [30, 31] (Figure 6A, left). Importantly, degree differs from membership diversity by being independent of community (regions have functional connections both within and between communities). As in the previous section, the distribution of degree across regions was plotted for each condition (BOLD, ${\text{Ca}}_{slow}^{2+}$, and ${\text{Ca}}_{fast}^{2+}$) (Figure 6A, right), the spatial distribution was displayed on the cortex (Figure 6B), and (Pearson) correlation was used to measure agreement between conditions (Figure 6C).

Across conditions, degree distributions showed weak similarity (Figure 6A, right). As expected, ${\text{Ca}}_{slow}^{2+}$ and ${\text{Ca}}_{fast}^{2+}$ were more similar to one another than to BOLD, and ${\text{Ca}}_{slow}^{2+}$ was more like BOLD than ${\text{Ca}}_{fast}^{2+}$. BOLD and both Ca^2+^ measures showed differences at the low-extreme (near zero) as well as across the range: Ca^2+^ having fewer low-mid degree regions, and more high-degree regions than BOLD. Intermodal differences were more pronounced when we looked at the spatial distribution of degree (Figure 6B), and the (Pearson) correlation of degree across conditions (Figure 6C). As for membership diversity, degree showed a consistent spatial pattern across Ca^2+^ conditions (Figure 6B), and was highly correlated: *r* = 0.87 ± 0.08 (Figure 6C, right). Unlike membership diversity, BOLD and both Ca^2+^ degree measures showed opposing spatial patterns (Figure 6B), and were negatively correlated with BOLD: between BOLD and ${\text{Ca}}_{slow}^{2+}$ : *r* = −0.29 ± 0.16, and BOLD and ${\text{Ca}}_{fast}^{2+}$ : *r* = −0.46 ± 0.14 Figure 6C (left and middle). To account for differences based solely on the magnitude/variability of degree values, we computed percentile maps by calculating t-statistics followed by rank-ordering [33] and found the patterns to be unchanged (not shown). Further, differences across modalities persisted across edge thresholds and changes in data preprocessing steps (Figure S7).

### Different entropy-degree relationships across modalities

**Figure 7:**
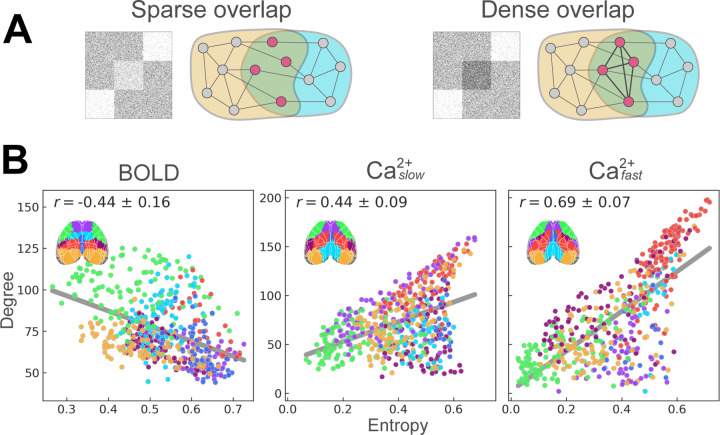
Entropy-degree relationships across modalities. **(A)** Illustrated examples of sparse and dense overlapping organization. Figure inspired by Yang and Leskovec [65]. **(B)** Entropy versus degree for BOLD (left), ${\text{Ca}}_{slow}^{2+}$ (middle), and ${\text{Ca}}_{fast}^{2+}$ (right). Each point corresponds to a brain region, color coded by their disjoint network assignment (inset; see last column in Figure 2C). See also Figure S6.

How a given brain region affiliates across multiple networks (as indexed by membership diversity/entropy) is closely linked to its roles as an integrative and/or coordination hub [34]. Furthermore, membership diversity and degree are measures that, when combined, can further uncover brain organization [32, 33]. In particular, regions with low entropy and high degree have few inter-network functional connections (low entropy) and many intra-network functional connections (high degree), and can be thought of as *provincial* hubs [33, 63, 64]. Regions with high entropy and low degree have few functional connections but link many networks, and can be conceptualized as *connector* hubs. Inspired by the framework developed by Yang and Leskovec [65], such organization reveals what can be called “sparse” network overlap (Figure 7A, left). Finally, regions with high entropy and high degree interlink many networks via an organization that can be called “dense” overlap (Figure 7A, right). To determine cortical functional organization based on these measures, we visualized region entropy-degree relationships for each condition (BOLD, ${\text{Ca}}_{slow}^{2+}$ and ${\text{Ca}}_{fast}^{2+}$) (Figure 7B) color-coded by disjoint network assignment (Figure 7B; inset).

Entropy and degree were inversely (Pearson) correlated for BOLD (*r* = −0.44 ± 0.16; Figure 7B, left). This pattern was partly driven by a concentration of regions showing sparse overlap (lower right quadrant) with connector hubs present in most networks, alongside two networks (overlapping with OC-3 and to a lesser extent OC-2; Figure 2C), that included regions with a more provincial hub characterization (upper left quadrant). In contrast, entropy and degree were positively correlated for ${\text{Ca}}_{slow}^{2+}$ (*r* = 0.44 ± 0.09), and ${\text{Ca}}_{fast}^{2+}$ (*r* = 0.69 ± 0.07) (Figure 7B, middle and right). Like BOLD (but to a lesser extent), ${\text{Ca}}_{slow}^{2+}$ results included regions with sparse overlap (lower right quadrant); these overlapped with OC-6, and to a lesser extent OC-1 and OC-7 (Figure 2C). However, unlike BOLD, there were regions with high entropy (densely overlapping regions; upper right quadrant), as well as regions with low overall functional connectivity (lower left quadrant). This pattern was more pronounced in the ${\text{Ca}}_{fast}^{2+}$ results where fewer regions exhibited sparse overlap (lower right quadrant; Figure 7B, right). Together, these results uncovered distinct functional cortical organization observed with BOLD, ${\text{Ca}}_{slow}^{2+}$, and ${\text{Ca}}_{fast}^{2+}$, such that Ca^2+^ signals expressed patterns of denser overlap not captured by BOLD signals.

## Discussion

We used recently developed simultaneous wide-field Ca^2+^ and fMRI-BOLD acquisition to characterize the functional network architecture of the mouse cortex. The spatial organization of large-scale networks discovered by both modalities showed many similarities, with some temporal frequency dependence (BOLD networks were generally more similar to ${\text{Ca}}_{slow}^{2+}$ than ${\text{Ca}}_{fast}^{2+}$). Functional connectivity interrogated using a mixed-membership algorithm, instead of traditional disjoint approaches, confirmed the hypothesis that mouse cortical networks exhibit substantial overlap when either BOLD or Ca^2+^ signals were considered. Further, despite the considerable agreement, we also uncovered important differences in organizational properties across signal modalities.

Previous multimodal studies comparing cortical functional organization via concurrent GCaMP6 Ca^2+^ and hemoglobin-sensitive imaging have predominantly employed seed-based analyses [3, 16, 66]. Such work provides information on how one or a limited set of *a priori* regions are functionally related to other areas but does not reveal how all regions are interrelated, which was the goal of the present work. A few studies using optical imaging have gone beyond seed-based analysis; however, the number of identified networks in these studies was limited. For example, Vanni and colleagues [4] investigated cortical networks in GCaMP6 mice and reported 3 networks based on slow temporal frequencies (< 1 *Hz*) and two based on faster temporal frequencies (3 *Hz*) (see also [5]). Here, when we decomposed the cortex into 3 networks, we observed visual (OC-2) and somatomotor (OC-3) networks and a network that overlapped with territories possibly linked to the default network (OC-1) [49, 51–53] (Figure 2A). At this coarse scale, our results agreed with Vanni et al. [4] and other seed-based approaches [3, 16, 37, 51]. Importantly, we sought to determine functional organization at finer spatial levels, too. With 7 networks, we still observed visual (OC-2) and somatomotor (OC-3) networks, now together with a finer decomposition of other cortical systems (Figure 2C). Overall, our analyses reproduced previous observations at a coarse scale but characterized a more fine-grained decomposition of cortical functional organization.

Next, we quantified the concordance between BOLD and Ca^2+^ networks. Overall, collapsing across networks, outcomes from ${\text{Ca}}_{slow}^{2+}$ (BOLD-frequency matched) were more similar than ${\text{Ca}}_{fast}^{2+}$ to BOLD, as expected [1–3, 17, 40–42]. However, when networks were characterized separately, three scenarios emerged: (1) Low and high frequency Ca^2+^ signals both manifested networks that were also recovered by BOLD (e.g., OC-1–OC-4); (2) Low relative to high frequency Ca^2+^ networks were a better match to their BOLD counterparts (e.g., OC-5 and OC-6); and (3) Networks that were dissimilar across modalities regardless of Ca^2+^ temporal frequency (e.g., OC-7). This observation should be qualified by the finding that ${\text{Ca}}_{slow}^{2+}$ and ${\text{Ca}}_{fast}^{2+}$ results were in close agreement. Overall, linking the functional organization obtained with BOLD to slow Ca^2+^ signals is not fully supported by our findings. In particular, the proposal that different bands capture distinct neurophysiological properties [67] was not supported for the large-scale system organization uncovered in the present work.

We quantified whether networks discovered for each condition (BOLD, ${\text{Ca}}_{slow}^{2+}$, and ${\text{Ca}}_{fast}^{2+}$) showed significant overlapping structure. This was accomplished by examining the distribution of membership values and by quantifying the number of networks each region “belonged to”. Notably, our algorithm detects disjoint organization in synthetic data, and the robustness of our findings was tested using a range of parameters (Table 1). Without exception, across conditions, parameter choices, and for all networks, we observed evidence of significant overlapping organization. On average, slightly over half of brain regions were affiliated with more than one network. Critically, although the extent of network overlap was largest for BOLD, it was also pronounced in Ca^2+^ data regardless of temporal frequency. These results lend strong support to the validity of overlapping organization in the human brain discovered with BOLD [25–28].

Properties of network overlap, membership diversity (entropy) and degree, were quantified at the region-level and compared across conditions (BOLD, ${\text{Ca}}_{slow}^{2+}$, and ${\text{Ca}}_{fast}^{2+}$ ). As expected, Ca^2+^ results showed low diversity in sensorimotor regions relative to areas that have been implicated in multiple processes and have widespread anatomical connections such as the posterior parietal cortex (which includes higher-order visual areas) [68, 69]. Despite showing a positive correlation with Ca^2+^ results, BOLD membership diversity measures showed some peculiarities. Specifically, in contrast to Ca^2+^, the posterior parietal cortex exhibited low diversity, while somatosensory areas exhibited high diversity. This was unexpected given that these regions are not known to be functionally diverse (again, Ca^2+^ data produced the anticipated outcome). Overall, these discrepancies do not disrupt a positive correlation between BOLD and Ca^2+^ but raise questions about the extent to which the two imaging techniques are capturing the same phenomena. Further, the story became more complicated when we examined region degree, which showed a negative correlation between BOLD and Ca^2+^ measurements with clearly different spatial patterns.

The relationship between entropy and degree helped to uncover additional properties of cortical functional organization. For BOLD, membership diversity and degree were inversely correlated, a pattern indicative of “sparse overlap” alongside some networks that included “provincial” hubs. Notably, this pattern has been observed in human BOLD data [32, 34]. In contrast, Ca^2+^ data exhibited a positive correlation (that was more pronounced for ${\text{Ca}}_{fast}^{2+}$), suggestive of “dense overlap” alongside regions showing few connections. Together, these results indicated that BOLD and Ca^2+^ capture distinct forms of overlapping network organization, with Ca^2+^ signals particularly able to uncover a “diverse club” of regions (**Bertolero) that are densely overlapping (*Dense/sparse paper), reminiscent of the “communication core” organization of structural connections in nonhuman primates (**Modha and Singh, 2010; Markov/Kennedy 2013 Science paper).

It is possible that some of the discrepancies between BOLD and Ca^2+^ results stemmed from Ca^2+^ signals originating from excitatory neurons, while the BOLD signal is cell-type agnostic. Despite excitatory neurons being the most populous cell type in the cortex, it is still unclear to what extent the activity of other cell populations (inhibitory neurons or astrocytes [70]) or vascular effects [7, 8, 71, 72] ) influence the BOLD signal. This will be explored in our future work utilizing the methods established here. Another important consideration is our use of anesthesia. Due to the challenges of imaging awake mice [73], especially head motion, we opted to use low levels of anesthesia. Head motion systematically alters the correlation structure of functional data and was therefore of particular concern in our analyses [74, 75] (Methods). However, the effects of anesthesia on brain activity and neurovascular coupling are complex and may vary by region, anesthetic agent, and dose [66, 76–78]. Our future studies will evaluate how functional networks, and their properties, differ between awake and anesthetized animals. Notably, the effects of brain state on functional organization were minimized given that the simultaneous nature of our multimodal data, given the same “ground truth” brain activity underlies the results from each modality. Thus, moment-to-moment brain-state differences were not driving factors behind our findings. However, the analysis methods we used do not strictly require simultaneous data collection. Further, although the findings reported in the main text were at the group level, our highly-sampled dataset allowed network organization to be determined at the level of the individual, lending considerable strength to our group-level findings, and underscoring the translational potential of our approach [79]. Exploring individualized network properties and exploiting the simultaneous nature of these data further will be a focus of future work.

Processing and analyzing multimodal data entails making several parameter choices that potentially affect outcome measures. In particular, network overlap could be inflated by spatial misalignment. We took great care in co-registering our data and optimizing our parameter set (from the Advanced Normalization Tools package [80]). Further, issues of misalignment were considerably reduced by estimating network measures at the level of runs and combining values subsequently. Thus, modest misalignment after registration did not inflate the overall evaluation of overlap (Methods). In addition, the quantification of membership strength was applied to values that were thresholded based on statistical significance. We also used relatively sparse graphs (15% density in the main text), such that only the strongest correlations were considered; further analyses that quantified the extent of overlap considered only membership values that statistically exceeded 1/7 (for the 7-network solution). We also probed the effects of parameter changes (Table 1) and found that our results were qualitatively robust.

In conclusion, we employed novel simultaneous wide-field Ca^2+^ imaging and BOLD in a highly sampled group of mice expressing GCaMP6f in excitatory neurons to determine the relationship between large-scale networks discovered by the two techniques. Our findings demonstrated that (1) most BOLD networks were detected via Ca^2+^ signals. (2) Considerable overlapping—as opposed to disjoint—network organization was recovered by both modalities. (3) The large-scale functional organization determined by Ca^2+^ signals at low temporal frequencies (0.01 − 0.5*Hz*), relative to high frequencies (0.5 − 5*Hz*), was more similar to those recovered with BOLD; yet, qualitative differences were also observed. (4) Key differences were uncovered between the two modalities in the spatial distribution of membership diversity and the relationship between region entropy (i.e., network affiliation diversity) and degree. Together these findings uncovered a distinct overlapping network phenotype across modalities. In sum, this work revealed that the mouse cortex is functionally organized in terms of overlapping large-scale networks that are observed with BOLD, lending fundamental support for the neural basis of such a property, which is also observed in human subjects. The robust differences which were uncovered demonstrate that Ca^2+^ and BOLD also capture some complementary features of brain organization. Future work exploring these commonalities and differences, using the simultaneous multimodal acquisition used here, promises to help uncover how large-scale networks are supported by underlying brain signals in health and disease.

## Materials and methods

### Experimental model and subject details

All procedures were performed in accordance with the Yale Institutional Animal Care and Use Committee and are in agreement with the National Institute of Health Guide for the Care and Use of Laboratory Animals. All surgeries were performed under anesthesia.

#### Animals

Mice were group housed on a 12-hour light/dark cycle. Food and water were available *ad libitum*. Cages were individually ventilated. Animals were 6–8 weeks old, 25–30g, at the time of the first imaging session. Animals (SLC, Slc17a7-cre/Camk2α-tTA/TITL-GCaMP6f also known as Slc17a7-cre/Camk2α-tTA/Ai93) were generated from parent 1 (Slc17a7-IRES2-Cre-D) and parent 2 (Ai93(TITL-GCaMP6f)-D;CaMK2a-tTA). Both were on a C57BL/6J background. To generate these animals, male CRE mice were selected from the offspring of parents with different genotypes, which is necessary to avoid leaking of CRE expression. Animals were originally purchased from Jackson labs.

#### Head-plate surgery

All mice underwent a minimally invasive surgical procedure enabling permanent optical access to the cortical surface (previously described here: [1]). Briefly, mice were anesthetized with 5% isoflurane (70/30 medical air/O_2_) and head-fixed in a stereotaxic frame (KOPF). After immobilization, isoflurane was reduced to 2%. Paralube was applied to the eyes to prevent dryness, meloxicam (2 mg/kg body weight) was administered subcutaneously, and bupivacaine (0.1%) was injected under the scalp (incision site). Hair was removed (NairTM) from the surgical site and the scalp was washed with betadine followed by ethanol 70% (three times). The scalp was removed along with the soft tissue overlying the skull and the upper portion of the neck muscle. Exposed skull tissue was cleaned and dried. Antibiotic powder (Neo-Predef) was applied to the incision site, and isoflurane was further reduced to 1.5%. Skull-thinning of the frontal and parietal skull plates was performed using a hand-held drill (FST, tip diameter: 1.4 and 0.7 *mm*). Superglue (Loctite) was applied to the exposed skull, followed by transparent dental cement (C&B Metabond^®^, Parkell) once the glue dried. A custom in-house-built head plate was affixed using dental cement. The head-plate was composed of a double-dovetail plastic frame (acrylonitrile butadiene styrene plastic, TAZ-5 printer, 0.35 *mm* nozzle, Lulzbot) and a hand-cut microscope slide designed to match the size and shape of the mouse skull. Mice were allotted at least 7 days to recover from head-plate implant surgery before undergoing the first of three multimodal imaging sessions.

### multimodal image acquisition

All mice, *N* = 10, underwent 3 multimodal imaging sessions with a minimum of 7 days between acquisitions. All animals underwent all imaging sessions. None were excluded prior to the study end-point. Data exclusion (based on motion etc.) is described below. During each acquisition, we simultaneously acquired fMRI-BOLD and wide-field Ca^2+^ imaging data using a custom apparatus and imaging protocol we have described previously [1]. Functional MRI data were acquired on an 11.7 *T* system (Bruker, Billerica, MA), using ParaVision version 6.0.1 software. During each imaging session, 4 functional resting-state runs (10 min each) were acquired. In addition, 3 runs (10 mins each) of unilateral light stimulation data were acquired. These data are not used in the present study. Structural MRI data were acquired to allow both multimodal registration and registration to a common space. Mice were scanned while lightly anesthetized (0.5 − 0.75% isoflurane in 30/70 O_2_/medical air) and freely breathing. Body temperature was monitored (Neoptix fiber) and maintained with a circulating water bath.

#### Functional MRI

We employed a gradient-echo, echo-planar-imaging sequence with a 1.0 sec repetition time (TR) and 9.1 ms echo time (TE). Isotropic data (0.4 *mm* × 0.4 *mm* × 0.4 *mm*) were acquired along 28 slices providing near whole-brain coverage.

#### Structural MRI

We acquired 4 structural images for multimodal data registration and registration to a common space. (1) A multi-spin-multi-echo (MSME) image sharing the same FOV as the fMRI data, with a TR/TE of 2500/20 *ms*, 28 slices, two averages, and a resolution of 0.1 *mm* × 0.1 *mm* × 0.4 *mm*. (2) A whole-brain isotropic (0.2 *mm* × 0.2 *mm* × 0.2 *mm*) 3D MSME image with a TR/TE of 5500/20 *ms*, 78 slices, and two averages. (3) A fast-low-angle-shot (FLASH) time-of-flight (TOF) angiogram with a TR/TE of 130/4 *ms*, resolution of 0.05 *mm* × 0.05 *mm* × 0.05 *mm* and FOV of 2.0 *cm* × 1.0 *cm* × 2.5 *cm* (positioned to capture the cortical surface). (4) A FLASH image of the angiogram FOV, including four averages, with a TR/TE of 61/7 *ms*, and resolution of 0.1 *mm* × 0.1 *mm* × 0.1 *mm*.

#### Wide-field fluorescence Ca^2+^ imaging

Data were recorded using CamWare version 3.17 at an effective rate of 10 *Hz*. To enable frame-by-frame background correction, cyan (470/24, Ca^2+^-sensitive) and violet (395/25, Ca^2+^-insensitive) illumination (controlled by an LLE 7Ch Controller from Lumencor) were interleaved at a rate of 20 *Hz*. The exposure time for each channel (violet and cyan) was 40 *ms* to avoid artifacts caused by the rolling shutter refreshing. Thus, the sequence was: 10 *ms* blank, 40 *ms* violet, 10 *ms* blank, 40 *ms* cyan, and so on. The custom-built optical components used for in-scanner wide-field Ca^2+^ imaging have been described previously [1].

### Image preprocessing

#### multimodal data registration

All steps were executed using tools in BioImage Suite (BIS) specifically designed for this purpose [1]. For each animal, and each imaging session, the MR angiogram was masked and used to generate a view that recapitulates what the cortical surface would look like in 2D from above. This treatment of the data highlights the vascular architecture on the surface of the brain (notably the projections of the middle cerebral arteries, MCA) which are also visible in the static wide-field Ca^2+^ imaging data. Using these and other anatomical landmarks, we generated a linear transform that aligns the MR and wide-field Ca^2+^ imaging data. The same static wide-field image was used as a reference for correcting motion in the time series. To register the anatomical and functional MRI data, linear transforms were generated and then concatenated before being applied.

Data were registered to a reference space (CCFv3, [35]) using isotropic whole-brain MSME images via affine followed by non-linear registration (ANTS, Advanced normalization tools; [80]). The histological volume in CCFv3 was used because of a better contrast match with MRI images. The goodness of fit was quantified using mutual information and a hemispheric symmetry score that captured the bilateral symmetry of major brain structures. A large combination of registration hyperparameters was explored, and the top 10 fits per animal were selected. The best transformation out of this pool was selected for each animal by visual inspection.

#### Fluorescence Ca^2+^ imaging data preprocessing

We have previously described all steps in this pipeline [43]. Briefly, the raw signal was split between GCaMP-sensitive and GCaMP-insensitive imaging frames. Spatial smoothing with a large kernel (16-pixel kernel, median filter) was applied to reduce and/or remove focal artifacts (e.g., dust or dead pixels from broken fibers). Focal artifacts do not move with the subject and can bias motion correction. Motion correction parameters were estimated on these data using normalized mutual information. Rigid image registration was performed between each imaging frame in the time series and the reference frame. Registration parameters were saved, and the large kernel-smoothed images were discarded. Modest spatial smoothing (4-pixel kernel, median filter) was applied to the raw data, and these data were motion corrected by applying the parameters estimated in the previous step. Data were down-sampled by a factor of two in both spatial dimensions, which yielded a per pixel spatial resolution of 50 × 50 *µm*^2^ (original was 25 × 25 *µm*^2^). Photo bleach correction was applied to reduce the exponential decay in fluorescence at the onset of imaging [81]. The fluorophore-insensitive time series were regressed from the fluorophore-sensitive time series. The first 50 seconds of data were discarded due to the persistent effects of photo-bleaching in the Ca^2+^ data. Data were band-pass filtered (3rd order Butterworth) between [0.01 − 0.5] *Hz* (${\text{Ca}}_{slow}^{2+}$), and high-pass filtered between [0.5 − 5.0] *Hz* (${\text{Ca}}_{fast}^{2+}$), and 15 time points (1.5*s* of data) were discarded from both beginning and the end of the time series to avoid filtering-related edge artifacts [75].

#### RABIES fMRI data preprocessing

For fMRI preprocessing, we used RABIES (Rodent automated BOLD improvement of EPI sequences) v0.4.2 [36]. We applied functional inhomogeneity correction N3 (nonparametric nonuniform intensity normalization; [82, 83]), motion correction (ANTS, Advanced normalization tools; [80]), and slice time correction, all in native space. A within-dataset common space was created by nonlinearly registering and averaging the isotropic MSME anatomical images (one from each mouse at each session), which was registered to the Allen CCFv3 reference space using a nonlinear transformation (see above).

For each run, fMRI data were motion corrected and averaged to create a representative mean image. Each frame in the time series was registered to this reference. To move the fMRI data to the common space, the representative mean image was registered to the isotropic structural MSME image acquired during the same imaging session. This procedure minimizes the effects of distortions caused by susceptibility artifacts [84]. Then, the three transforms — (1) representative mean to individual mouse/session isotropic MSME image, (2) individual mouse/session isotropic MSME image to within-dataset common space, and (3) within-dataset common space to out-of-sample common space –– were concatenated and applied to the fMRI data. Functional data (0.4mm isotropic) were upsampled to match anatomical MR image resolution (0.2mm isotropic). Registration performance was visually inspected and verified for all sessions. Motion was regressed (6 parameters). Data were high-pass filtered (3rd order Butterworth) between [0.01 − 0.5] *Hz*, and 15 time points (15*s* of data) were discarded from both the beginning and the end of the time series to avoid filtering-related edge artifacts. Average white matter and ventricle time courses were regressed.

#### Frame censoring

Data were scrubbed for motion using a conservative 0.1 *mm* threshold. High-motion frames were selected based on estimates from the fMRI time series and applied to both fMRI and Ca^2+^ data. Runs were removed from the data pool if half of the imaging frames exceed this threshold for a given run. In this dataset, 2 runs (or ∼ 1.7% of all runs) were removed for this reason. Additionally, 2 more runs were removed because they did not pass our quality control criteria.

### Parcellating the cortex into columnar regions of interest (ROI)

To create regions of interest (ROIs), we employed the Allen CCFv3 (2017) reference space [35] and used their anatomical delineations as our initial choice of ROIs. However, this led to poor performance (see supplemental discussion). Here, we introduce a novel spatially homogeneous parcellation of the mouse cortex that can be adopted for both 3D fMRI and 2D wide-field Ca^2+^ imaging data.

The procedure worked as follows. (1) We generated a cortical flatmap within the CCFv3 space using code published in ref. [85](https://github.com/AllenInstitute/mouse_connectivity_models). (2) We subdivided the left hemisphere into *N* regions via *k*-means clustering applied to pixel coordinates (for most analyses reported, *N* = 512). The right hemisphere was obtained by simple mirror-reversal to obtain a total of 2*N* regions. (3) Depth was added to the ROIs to obtain column-shaped regions. To do so, a path was generated by following streamlines normal to the surface descending in the direction of white matter (streamline paths were available at 10 *µm* resolution in CCFv3; see Figure 3F in [35]). Here, we chose ROI depths so that we included potential signals from approximate layers 1 to 4 (layer masks were obtained from CCFv3). Evidence from wide-field Ca^2+^ imaging suggests that signals originate from superficial layers but can extend into the cortex to some extent [15, 44, 86–88]. (4) Finally, ROIs were downsampled from 10 *µm* to 100 *µm* resolution. See Figure 1C.

After co-registration, ROIs were transformed from the CCFv3 space into each individual’s 3D and 2D anatomical spaces (see above). On average, ROIs had a size of 8 ± 3 voxels (3D, fMRI) and 48 ± 20 pixels (2D, Ca^2+^ ) in individual spaces (mean ± standard deviation).

### Functional network construction

Time series data were extracted and averaged from all voxels/pixels within an ROI in native space to generate a representative time series per ROI. For each modality, for each run, an adjacency matrix was calculated by applying Pearson correlation to time series data to each ROI pair. Next, we binarized the adjacency matrices by rank ordering the connection weights and maintaining the top 15%; thus, after binarization, the resulting graphs had a fixed density of *d* = 15% across runs and modalities. This approach aims to keep the density of links fixed across individuals and runs and better preserves network properties compared to absolute thresholding [89]. To establish the robustness of our results to threshold values, we also tested values of 10% to 25% in 5% increments.

### Finding overlapping communities

Overlapping network analysis was applied by using SVINET, a mixed-membership stochastic blockmodel algorithm [29, 90], which has been previously applied to human fMRI data by us [25] and other groups [27]. SVINET models the observed graph within a latent variable framework by assuming that the existence (or non-existence) of links between pairs of nodes can be explained by their latent community memberships. For binary adjacency matrix *A* and membership matrix π, the model assumes the conditional probability of a connection as follows

(1) $$p\left(A_{ij}|\pi_{i},\pi_{j}\right)\propto \sum_{k=1}^{K}\pi_{ik}\pi_{jk},$$

where *K* is the number of communities, and *A*_*ij*_ = 1 if nodes *i* and *j* are connected and 0 otherwise. Intuitively, pairs of nodes are more likely to be connected if they belong to the same community or to (possibly several) overlapping communities. More formally, SVINET assumes the following generative process

1. For each node, draw community memberships π_*i*_ ∼ Dirichlet(α)
2. For each pair of nodes *i* and *j*:
  - Draw community indicator *z*_*i*→ *j*_ ∼ π_*i*_
  - Draw community indicator *z*_*i*← *j*_ ∼ π _*j*_
  - Assign link between *i* and *j* if *z*_*i*→ *j*_ = *z*_*i*← *j*_.

Model parameters α are fit using stochastic gradient ascent [91, 92]. The algorithm was applied to data from each run using 500 different random seeds. Results across seeds were combined to obtain a final consensus for a run.

#### Aligning community results

Communities were identified in random order due to the stochastic nature of our algorithm. Maximum cosine similarity of the cluster centroids was used to match communities across calculations (runs or random seeds). For each run and random seed, membership vectors for all random seeds were submitted to *k*-means clustering (sklearn.cluster.KMeans) to determine *K* clusters (e.g., *K* = 7 for analyses with 7 communities). The similarity between the resulting cluster centroids was then established via cosine similarity, and the community matched based on maximum similarity. Formally, the outcome was membership matrix π (Figure 1E).

### Group results

Crucially, all measures were computed at the run level first before combining at the group level.

#### Membership matrices

This is what’s visualized in Figure 2A and C, Figure S1 and Figure S2A and C.

#### Thresholding membership values

To enhance the robustness of our estimates of network overlap, membership values were thresholded to zero if they did not pass a test rejecting the null hypothesis that the value was zero. After thresholding, the surviving membership values were rescaled to sum to 1. Thresholding was performed for each animal separately by performing a one-sample *t*-test and employing a false discovery rate of 5%. All results shown utilized this step, with the exception of figures which illustrate the spatial patterns of membership values (and do not estimate network overlap). Note that almost all (∼ 99%) memberships that did not reach significance had values in the range [0, 0.2].

#### Region functional diversity

Shannon entropy was applied to membership matrices for each run separately before averaging. That is, given a membership matrix π from a run, node entropies were computed to get an entropy estimate per node at the run level

(2) $$\text{(normalized) entropy of node }i\text{ at run level}=f\left(\pi_{i}\right)\equiv \sim h_{i}=-\sum_{k=1}^{K}\pi_{ik}\log\;\pi_{ik}/\log\;K\;,$$

where *K* = 7 is the number of communities. Entropy values were combined by averaging over runs to get the group-level estimates. This is what’s visualized in Figure 5B. Similarly, group averages were used to calculate the correlations between modalities in Figure 5C.

#### Computing distributions

Similar to above, distributions were computed for each run separately before combining at the group level. For example, consider *h*_*i*_ (entropy of node *i*) from a run. We computed percentage values using 20 bins of width 0.05 that covered the entire range of normalized entropy values [0, 1]. We then averaged over run-level histogram values to get group-level estimates shown in Figure 5A. Other distributions were computed in an identical way. Specifically, 57 bins of size 5 were used for Figure 6A, and 4 bins of size 0.2 were used for Figure 3B.

### Statistical analysis

#### Hierarchical bootstrapping

Statistical results were performed at the group level by taking into consideration the hierarchical structure of the data (for each animal, runs within sessions), which can be naturally incorporated into computational bootstrapping to estimate variability respecting the organization of the data [93]. For each iteration (total of 1, 000, 000), we sampled (with replacement) *N* = 10 animals, *N* = 3 sessions, and *N* = 4 runs, while guaranteeing sessions were yoked to the animal selected and runs were yoked to the session selected (Figure 1B). In this manner, the multiple runs were always from the same session, which originated from a specific animal. Overall, the procedure allowed us to estimate population-level variability based on the particular sample studied here. To estimate 95% confidence intervals, we used the bias-corrected and accelerated (BCa) method [94], which is particularly effective when relatively small sample sizes are considered (SciPy’s scipy.stats.bootstrap). See Figure 2E and F, Figure 3B, Figure 5C, Figure 6C, Figure 7B, Figure S2E and F, Figure S3, and Figure S6.

#### One-sample t-test

We used a one-sample *t*-test to define *belonging* in networks as a function of a threshold *µ*. The *t*-statistic for membership of node *i* in network *k* is given as

(3) $$t_{ik}=\frac{{\bar{\pi }}_{ik}-\mu }{SE_{ik}},$$

where ${\bar{\pi }}_{ik}$ is the group averaged membership of node *i* in network *k*, and *SE*_*ik*_ is the standard error estimated using hierarchical bootstrapping (see above). We calculated p values using *t*-statistics for all nodes and networks and declared a node a member of a network if its p-value reached significance *p* = 0.05. The results as a function of various *µ* are shown in Figure 4A. We applied Benjamini-Hochberg correction [95] using Python statsmodels’ implementation (statsmodels.stats.multitest.multipletests) to correct for multiple comparison.

#### LFR analysis

The following parameters need to be specified to generate a binary and overlapping LFR graph [62]. *N*, number of nodes; *k*, average degree; *µ*, topological mixing parameter; *t*_1_, minus exponent for the degree sequence; *t*_2_, minus exponent for the community size distribution; *C*_*min*_, minimum for the community sizes; *C*_*max*_, maximum for the community sizes; *ON*, number of overlapping nodes; *OM*, number of memberships of the overlapping nodes.

To match basic statistics of the real data with LFR graphs we set *N* = 542 (Figure 1D); and, for every run from each data modality, we calculated the average degree *k* and estimated *t*_1_ via an exponential fit to the degree distributions (scipy.stats). We set *t*_2_ = 0.1, *C*_*min*_ = 0.05 × *N* ≈ 27, *C*_*max*_ = 0.35 × *N* ≈ 190. For the fraction of overlapping nodes *ON*, we explored a wide range between 0 (disjoint) and 0.9 in incremental steps of 0.1. This yielded *ON* = 0 up to *ON* = 0.9 × *N* = 488. Finally, we used *OM* = 2 and 3. This results in a total of 20 LFR graphs per run, per data modality. We applied the community detection algorithm to LFR graphs in an identical way to the real data, but with fewer seeds (*N* = 10 compared to *N* = 500). The alignment procedure was performed in an identical way as described above. See Figure S5.

#### Permutation test

A paired permutation test was used to compare conditions in Figure 2E and F, Figure S2E and F, and Figure S3; and to perform a node-wise comparison across modalities in Figure 5D. We used SciPy’s implementation (scipy.stats.permutation_test) with *N* = 1, 000, 000 resamples. Holm–Bonferroni correction was applied to correct for multiple comparison [96].

## Supplementary Material

Supplement 1

## Figures and Tables

**Table 1:** To ensure the robustness of our findings, we explored a range of parameters and found that our results were qualitatively reproduced across all conditions.

Parameters explored in the present study	
Parameter	Values and figures
Number of networks	3 (Figure 2); 7 (Figures 3–7); and 20 (Figure S1)
Initial parcellation granularity	Fine (542 regions, Figure 1D); and Coarse (152 regions, Figure S2)
Network density	10–25% (Figure S3, Figure S7)
fMRI preprocessing pipeline	Figure S7A
