# Supplementary material for: Multimodal measures of spontaneous brain activity reveal both common and divergent patterns of cortical functional organization
Source: Res Sq. 2023 Apr 26:rs.3.rs-2823802. Preprint. [Version 1] doi: 10.21203/rs.3.rs-2823802/v1 (PMC10168440; doi:10.21203/rs.3.rs-2823802/v1)
Supplement: Supplement 1 [file NIHPPrs2823802v1-supplement-1.pdf]

Supplemental information

Supplemental discussion

Choosing number of communities

Clustering is in the eyes of the beholder (or the algorithm) [97]. Community detection is inherently ill-defined: algorithms do not find communities, what they do is *"define"* communities and later find them according to their definition. Here, we employed a mixed-membership stochastic blockmodel [46], where each community corresponds to a *latent functional role* (see [98] for an extensive review). Choosing a specific number of communities is thus equivalent to deciding how many functional roles (or clusters) best describe the observed graphs. In this sense, there is no *"true"* number of communities. Therefore, instead of focusing on a single result as a true decomposition, we explored network organization at different levels of granularity. We decided the number of communities empirically, using criteria such as bilateral symmetry. At the most coarse level, our $K = 3$ communities recapitulated previous seed-based reports in Ca^{2+} data and coarse fMRI-ICA findings. Our $K = 7$ decomposition was similar to previous fMRI-ICA reports [37, 50]. Overall, the highly bilateral nature of the resulting community structure (observed for up to $K = 20$ for Ca^{2+}) and their similarity to previous reports increased our confidence about our results.

Thresholding the graphs

The algorithm used in this study requires binary graphs as its input [29], which necessitates choosing an edge-filtering threshold. To mitigate this, we employed an approach known as proportional thresholding which is known to perform better than alternatives such as absolute thresholding [89]. In addition, theoretical work has shown that network topology is highly robust against different thresholds [99]. Here we reported results at a graph density of $d = 15\%$. To ensure the robustness of our findings to this arbitrary choice, we also explored densities going from $d = 10\%$ all the way up to $d = 25\%$ with incremental steps of 5%. Empirically, we found that our results were robust to the choice of edge density, as well as other hyperparameters. Overall, our data partially confirmed previous theoretical and simulation work that network topology is robust across a wide range of sparsity levels.

Region of interest (ROI) definition scheme

We started by using brain region masks from Allen Reference Atlas (ARA) as our initial choice of ROIs [35]. However, we observed some mismatch between functional parcellations and ARA parcellation. This is probably because ARA regions were delineated using various anatomical and structural criteria; but crucially, function was not one of them. In the present work, we introduced a new parcellation scheme illustrated in Figure 1C and D, which increased the robustness of our results. In conclusion, we found that spatially homogeneous ROIs worked well for the purpose of functional network construction, consistent with previous reports in humans [100].

Defining appropriate ROIs for our multimodal dataset was challenging because different modalities occupy spaces with different geometries. Namely, fMRI data is defined within a 3D volumetric space, while mesoscopic Ca^{2+} imaging data exists only on the 2D cortical surface. To address this, we started from the 2D space of cortical flatmap (Figure 1C; step I), which fits Ca^{2+} data well. We then added depth to obtain 3D volumetric ROIs (Figure 1C; step II), suitable for fMRI data. In the depth dimension, we included cortical layers 1 to 4. Crucially, our goal was not to obtain layer-specific results (BOLD resolution was 0.4 *mm* isotropic). Instead, we wanted to consider depths of the cortex that most likely contribute to

Ca^{2+} signal [15, 44, 86–88], which would render our network-level cross-modality comparisons more meaningful.

Comparing entropy to other measures of node diversity

Brain regions of high functional diversity are more likely to participate in multiple networks. This region-level property can be characterized using appropriate node centrality measures. Here, we took advantage of having access to continuous membership values and defined node entropy centrality (Figure 5). Another measure, *"participation coefficient"* [32–34, 63, 64], has also been commonly used to quantify a similar concept: a node's participation coefficient measures how well-distributed its links are among different communities. Large participation coefficient indicates higher amount of link diversity, which could be potentially related to high membership diversity. To understand the relationship between the two measures, we visualized their spatial patterns and found that entropy and participation coefficient maps were largely in agreement (Figure S6). The node-wise correlation between entropy and participation coefficient was $r = 0.70 \pm 0.09$, BOLD; $r = 0.77 \pm 0.12$, Ca^{2+}_{slow} ; $r = 0.47 \pm 0.28$, Ca^{2+}_{fast} .

It is worth noting that node entropy and participation coefficient are defined in very different ways. Entropy is computed from membership probability vectors within our overlapping framework; whereas, participation coefficient depends on how links are distributed across communities within a disjoint framework. Despite this, the two measures were highly correlated indicating that they probably capture similar underlying phenomena.

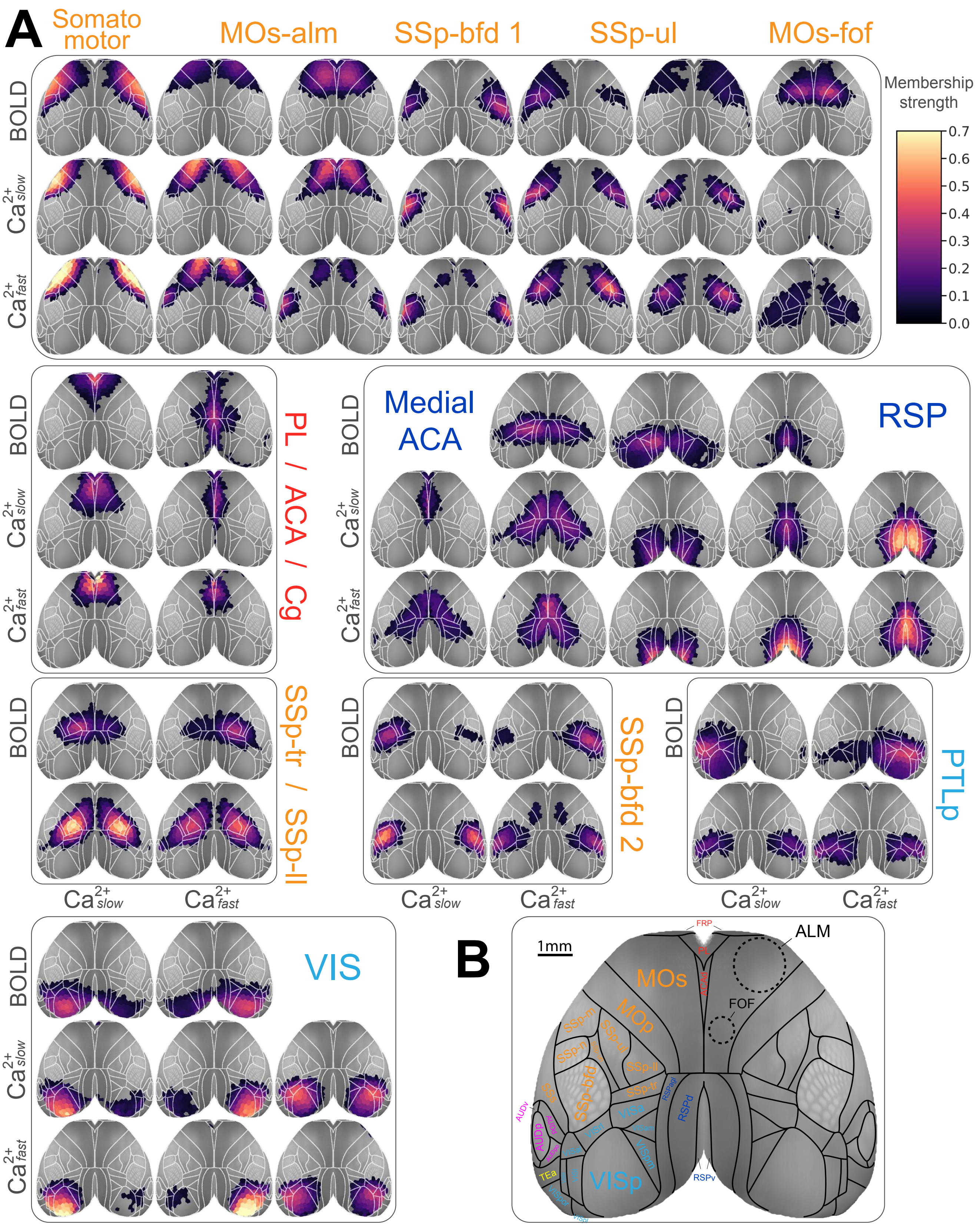

Figure S1: $K = 20$ decomposition. **(A)** Even at $K = 20$, most networks maintain their bilateral symmetry, especially for Ca^{2+} . A network centered around FOF appears as its own separate network for BOLD, similar to the $K = 7$ solution (top-right). In contrast, this network did not appear separately for Ca^{2+} , even at $K = 20$. Instead, FOF partially overlaps with a large medial Ca^{2+} network that spans parts of SSp-tr/ll. **(B)** Fine divisions of the cortical regions in Allen reference atlas, along with ALM and FOF (dashed lines). Label colors inspired from Figure 1F in ref. [101]. A complete list of abbreviations can be found in the original CCFv3 publication [35]. Compare with Figure 2 for $K = 3$ and $K = 7$ solutions.

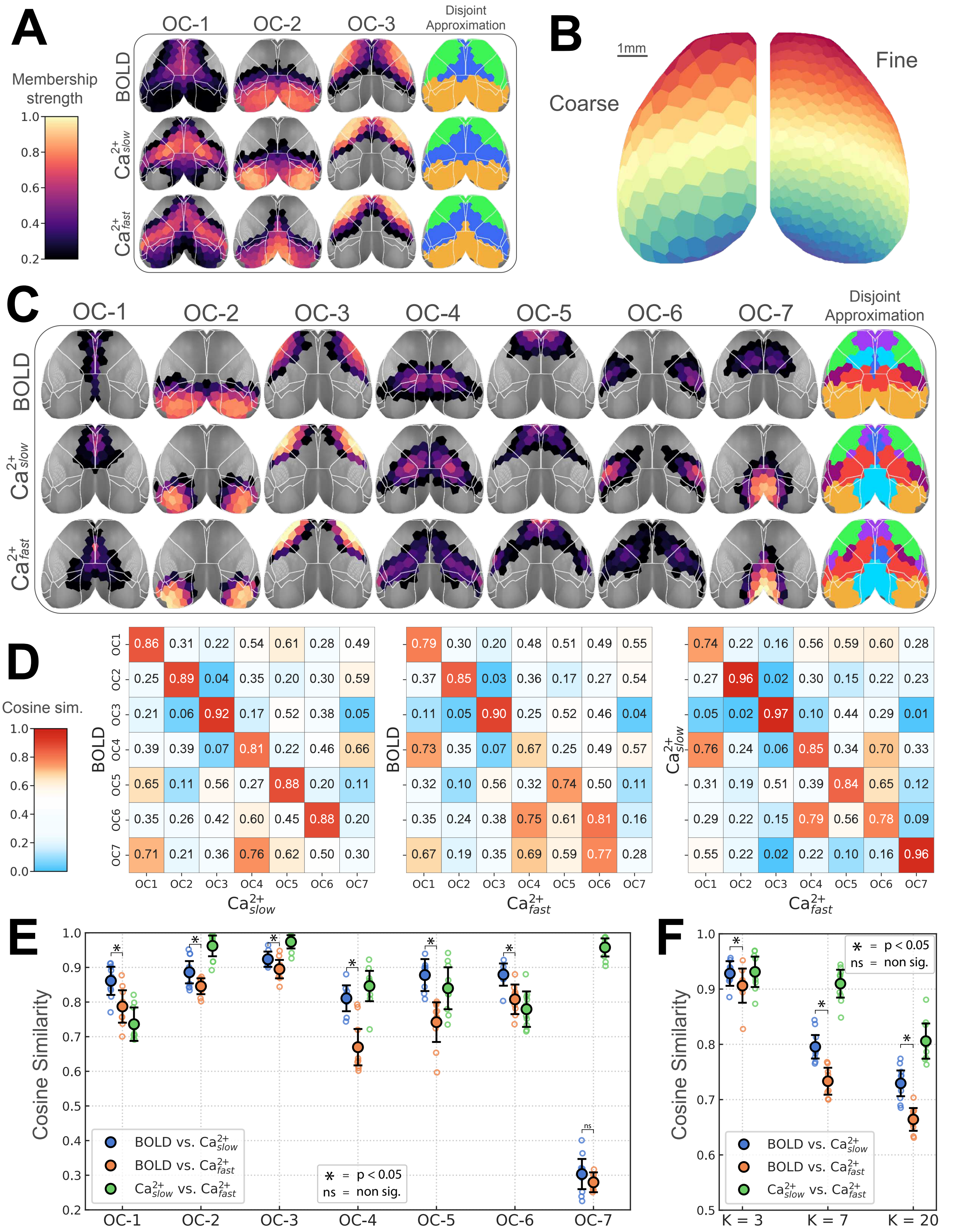

Figure S2: Network structure is robust to the choice of ROI granularity. **(A, C-F)** Similar to Figure 2. **(B)** Left, results with coarse ROIs are presented here; Right, fine ROIs were used for the main results. See also Figure S3.

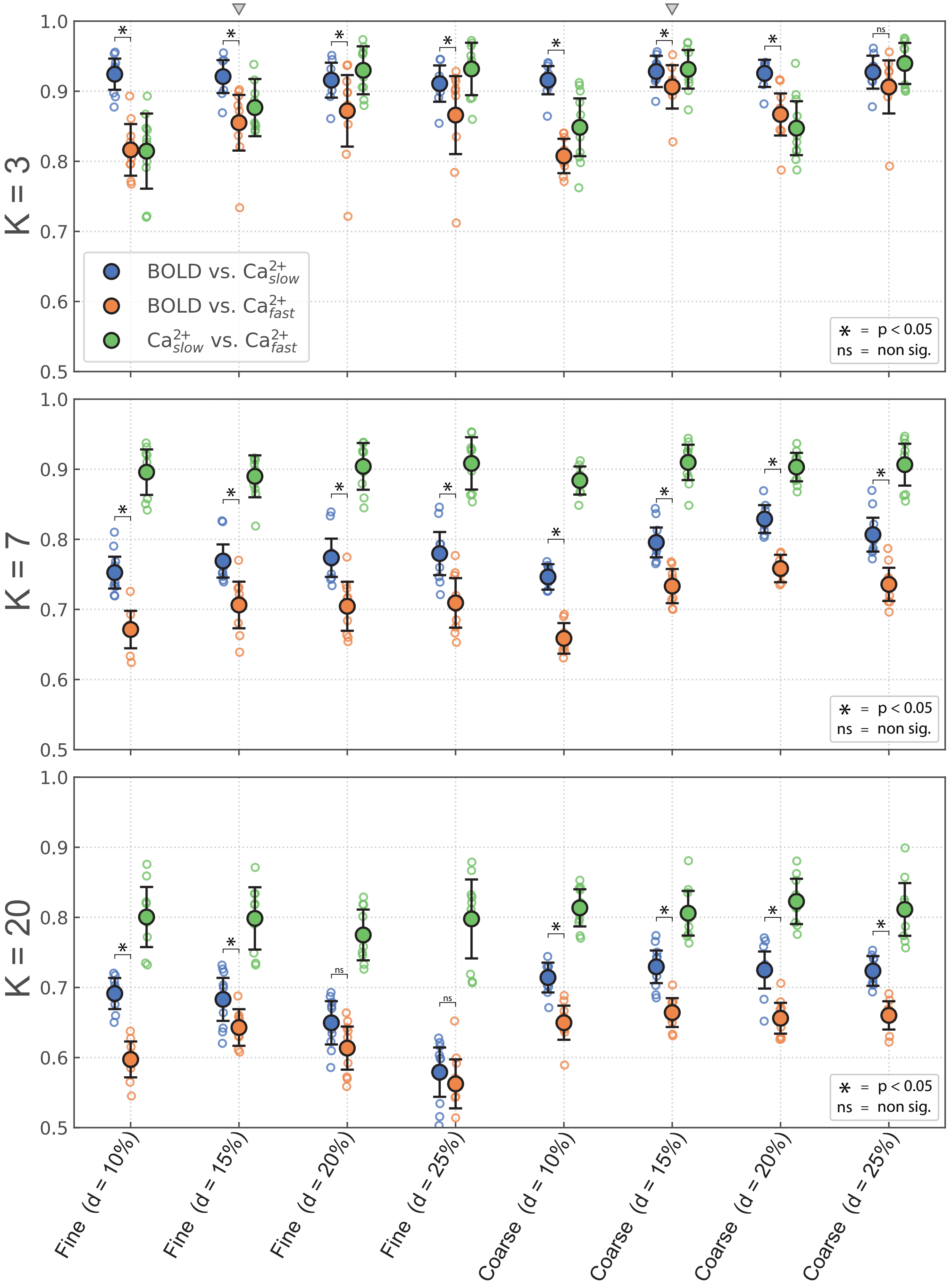

Figure S3: BOLD network organization is more similar to Ca^{2+}_{slow} than it is to Ca^{2+}_{fast} . This is a robust finding in the present study, as it is reproduced over a large combinatorial space of analysis conditions. The y-axis is cosine similarity and the x-axis corresponds to different conditions. See Figure S2B for a visual comparison of coarse versus fine ROIs. d is graph density after edge-filtering is applied. Small triangles indicate our choices for the main results: $d = 15\%$, fine ROIs. Permutation test, $p < 0.05$, Holm-Bonferroni corrected. Error bars show 95% confidence intervals, hierarchical bootstrap. See also Figure 2 and Figure S2.

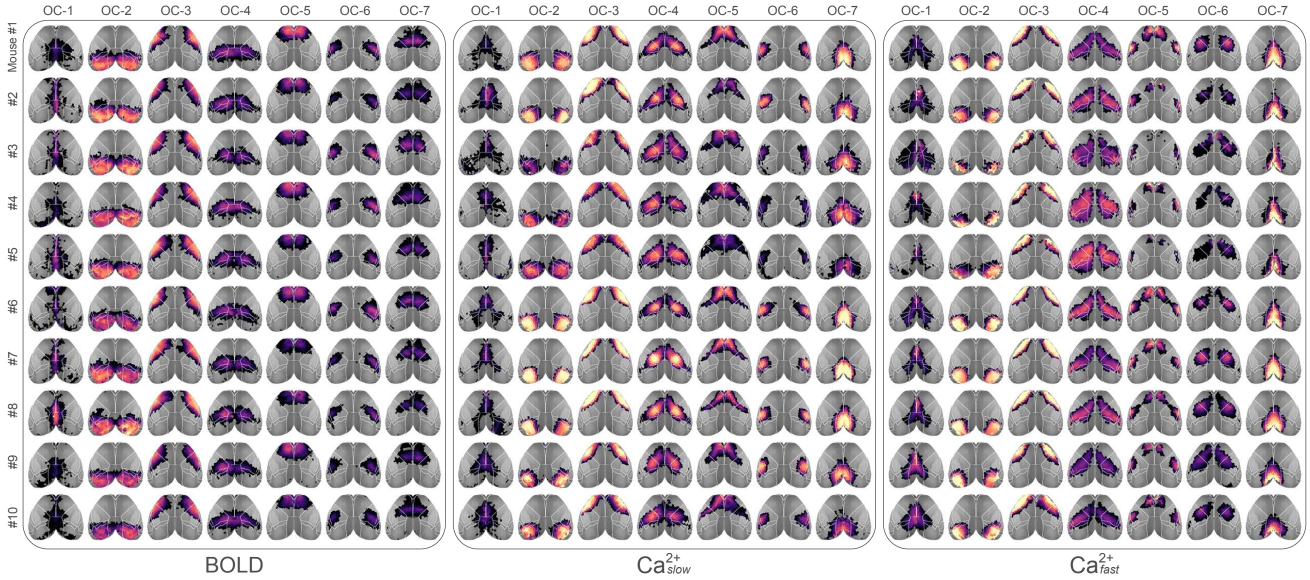

Figure S4: Overlapping networks at the level of individuals. Each mouse was highly sampled (Figure 1B), which allowed robust estimation of individualized networks. The high similarity of network structures across individuals is anticipated; however, there are still visible differences. Overall, the reproducibility of results at the individual level is notable and lends support to our group results. Compare with Figure 2C for group results.

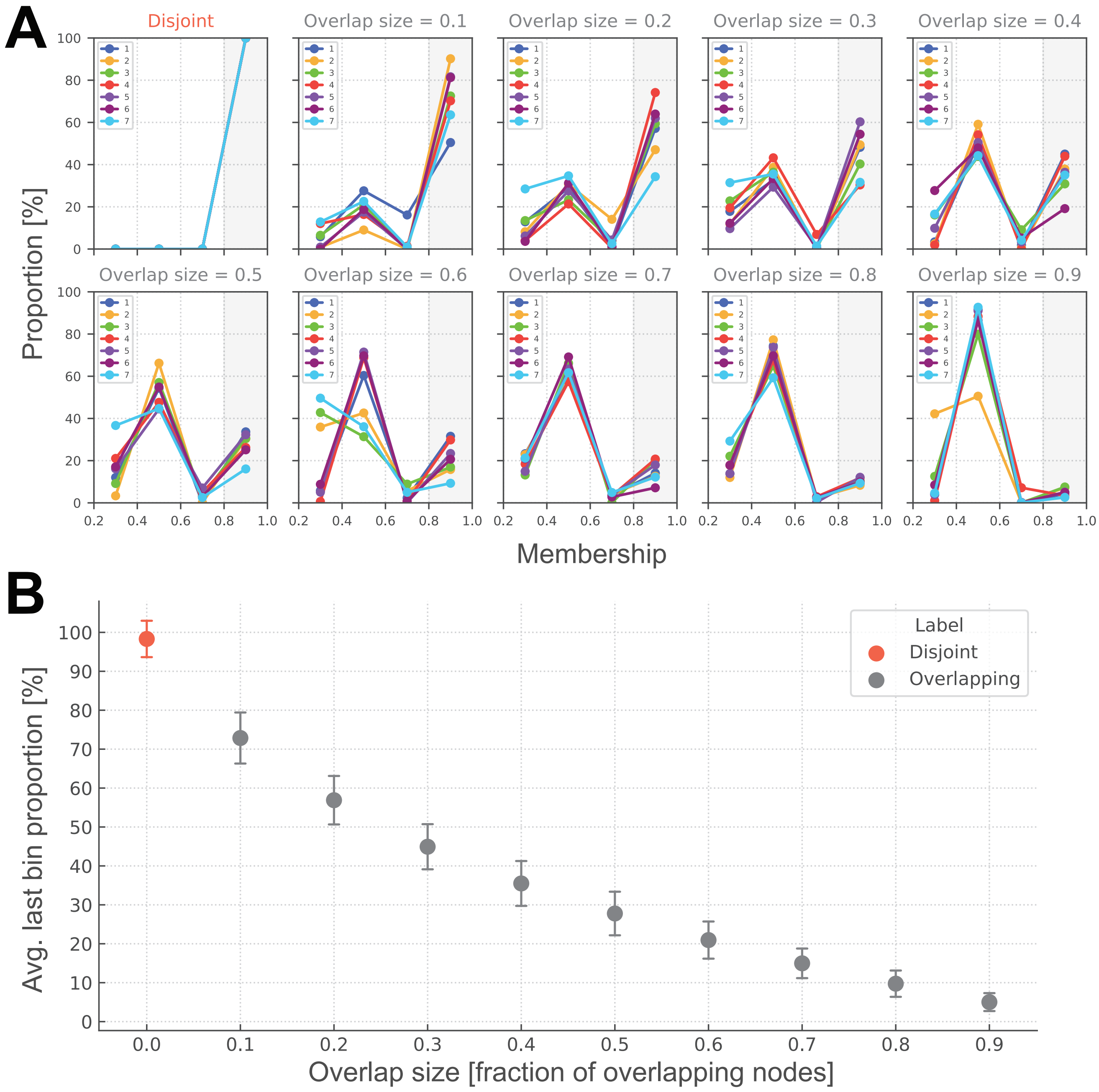

Figure S5: Verifying our analysis procedure using synthetic LFR graphs [62]. **(A)** To compute the distribution of node memberships, we divided the interval $(0.2, 1.0]$ into four bins of equal width. We chose this binning scheme because it allowed us to distinguish between disjoint and overlapping graphs: for disjoint graphs, the bin corresponding to strong membership values (i.e., last bin) had a proportion of 100%, and the proportions were 0% elsewhere. Overlap size, or fraction of overlapping nodes, is a tunable parameter in LFR graphs. Here we show how membership distributions change as a function of overlap size. **(B)** We averaged the last bin proportions across all communities to get a single statistic per graph. Its mean over thousands of simulated graphs is shown here. The average last bin proportion drops as overlap size increases, thus making it a reliable proxy for network overlap size. Error bars indicate standard deviation. Compare with Figure 3.

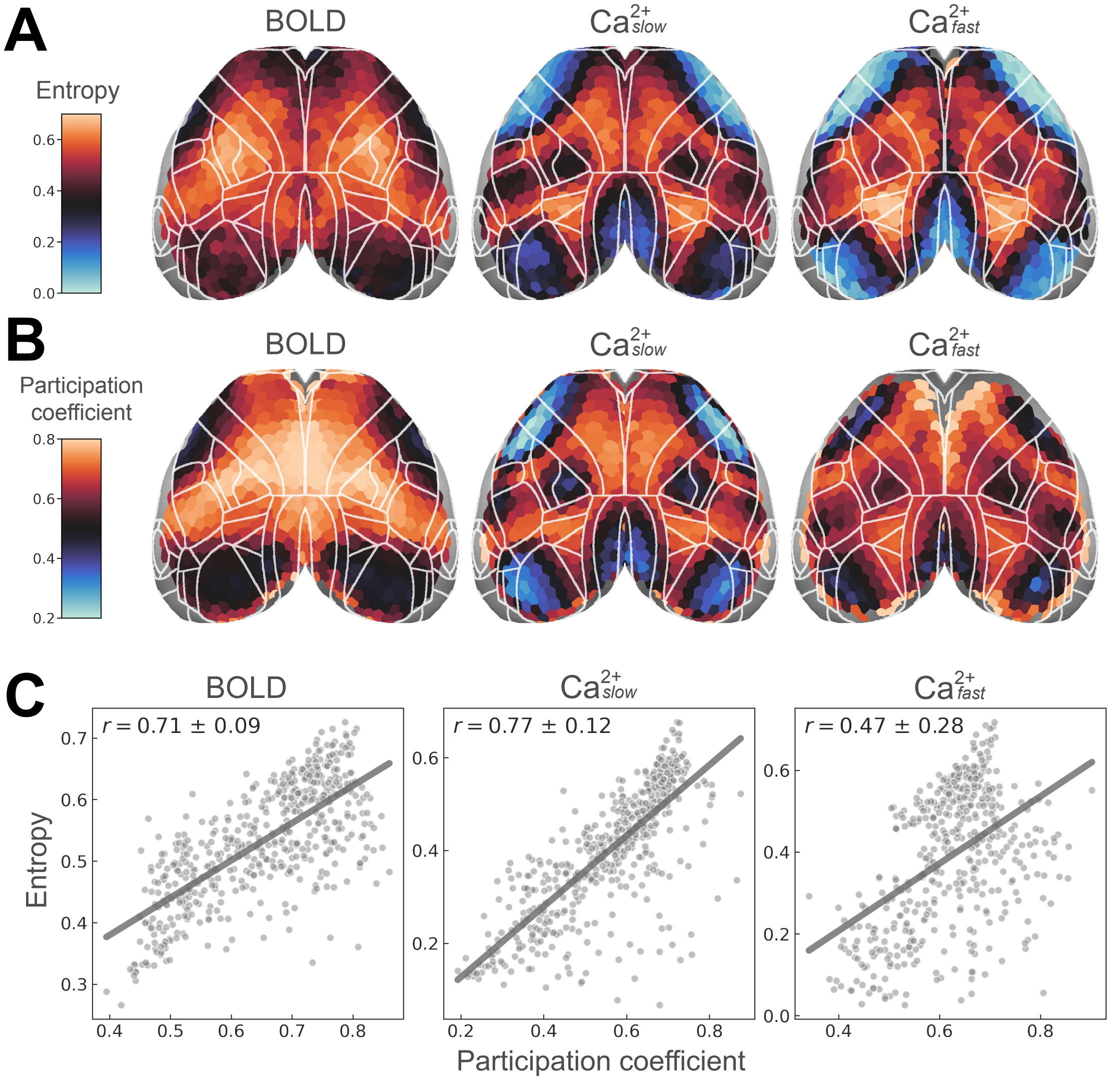

Figure S6: Entropy and participation coefficient uncover similar spatial patterns. **(A)** Here, we show entropy maps with actual values (compare with Figure 5B for the rank-ordered version). Note the positive shift in BOLD values. **(B)** Participation coefficient is commonly used to quantify how a node's links are distributed across (disjoint) communities [32–34, 63, 64]. To compute participation coefficients, we used the disjoint approximation obtained from taking the maximum membership of a given node (see the last column in Figure 2C). Note that for low-degree nodes with degree < 7, participation coefficient estimates become unreliable. For example, see frontal regions in Ca^{2+}_{fast} . **(C)** The two centrality measures are correlated, suggesting that they capture similar underlying phenomena. The concordance between these measures is most clearly visible for Ca^{2+}_{slow} , but also for BOLD to some extent.

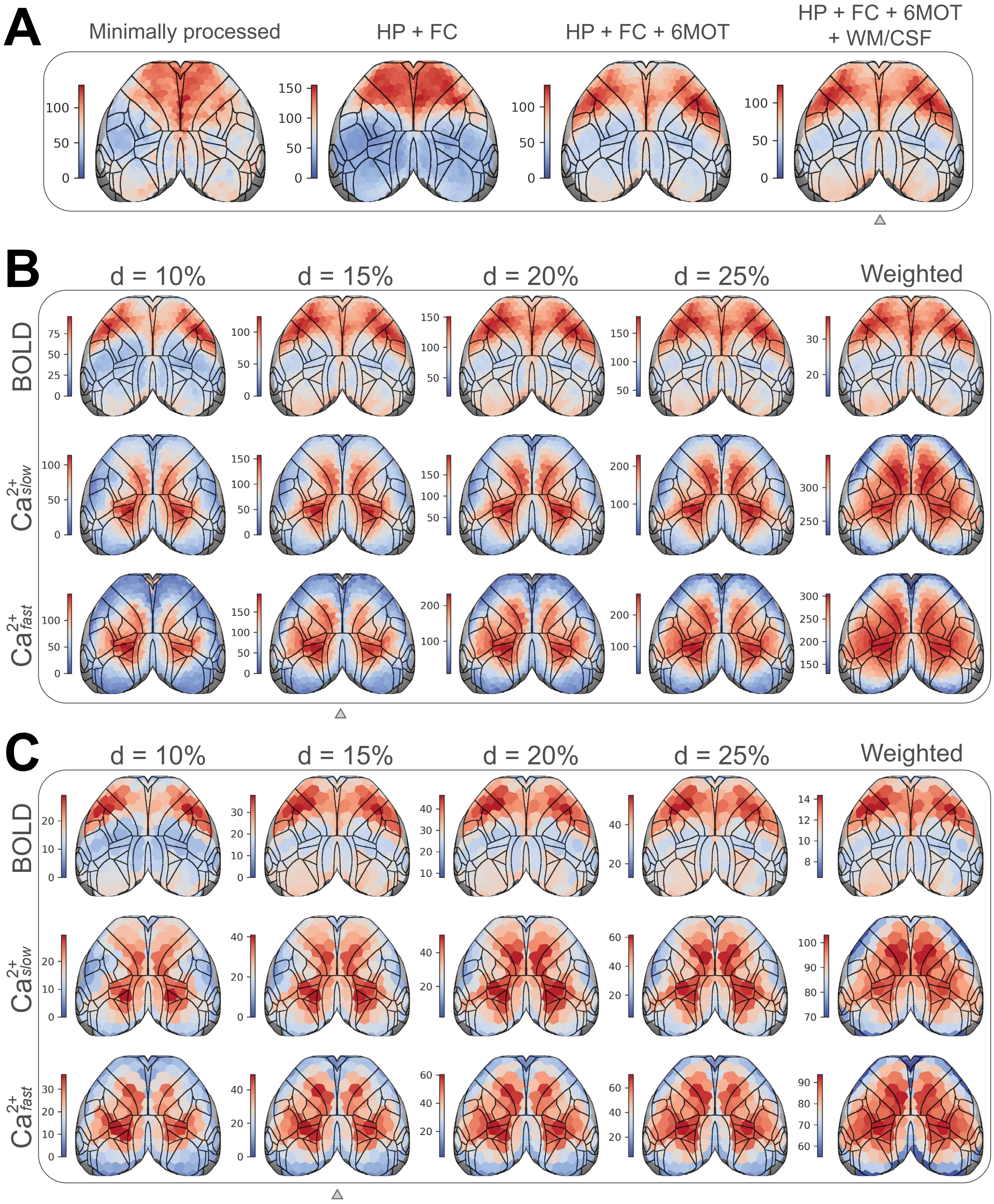

Figure S7: Dependence of degree centrality to preprocessing and analysis choices. **(A)** Spatial pattern of average node degree is somewhat altered depending on which BOLD preprocessing steps are used. Minimally processed, motion correction (rigid transformations) and detrending; HP, high-pass filtering (0.01 *Hz*); FC, frame censoring; 6MOT, motion regression (6 parameters); WM/CSF, average signal from white matter and ventricles regressed out. **(B)** Degree patterns are robust to the choice of edge filtering threshold. Difference thresholds result in different scales, but the spatial patterns remain relatively similar. **(C)** Similar to B but for a coarse parcellation (see Figure S2B). Small triangles indicate the pipeline and parameters used for the main results. Related to Figure 6.
